# A comparative analysis reveals the genomic diversity among 8 Muscovy duck populations

**DOI:** 10.1093/g3journal/jkae112

**Published:** 2024-05-24

**Authors:** Te Li, Yiming Wang, Zhou Zhang, Congliang Ji, Nengzhu Zheng, Yinhua Huang

**Affiliations:** State Key Laboratory of Farm Animal Biotech Breeding, College of Biology Sciences, China Agricultural University, No.2 Yuan Ming Yuan West Road, Hai Dian District, Beijing 100193, China; State Key Laboratory of Farm Animal Biotech Breeding, College of Biology Sciences, China Agricultural University, No.2 Yuan Ming Yuan West Road, Hai Dian District, Beijing 100193, China; National Key Laboratory for Swine Genetic Improvement and Production Technology, Jiangxi Agricultural University, Nanchang 330045, China; Technology Department (Research Institute) Livestock and Poultry Breeding Research Office, Wens Foodstuff Group Co. Ltd, Huineng North Road, Xincheng Town, Xinxing County, Yunfu City, Guangdong Province 527400, China; Institute of Animal Husbandry and Veterinary Medicine, Fujian Academy of Agricultural Sciences, Fuzhou 350013, China; State Key Laboratory of Farm Animal Biotech Breeding, College of Biology Sciences, China Agricultural University, No.2 Yuan Ming Yuan West Road, Hai Dian District, Beijing 100193, China

**Keywords:** SNPs, runs of homozygosity, homozygous-by-descent, selective sweep

## Abstract

The Muscovy duck (*Cairina moschata*) is a waterfowl indigenous to the neotropical regions of Central and South America. It has low demand for concentrated feed and strong adaptability to different rearing conditions. After introduced to China through Eurasian commercial trade, Muscovy ducks have a domestication history of around 300 years in the Fujian Province of China. In the 1990s, the commodity Muscovy duck breed “Crimo,” cultivated in Europe, entered the Chinese market for consumption and breeding purposes. Due to the different selective breeding processes, Muscovy ducks have various populational traits and lack transparency of their genetic background. To remove this burden in the Muscovy duck breeding process, we analyzed genomic data from 8 populations totaling 83 individuals. We identify 11.24 million single nucleotide polymorphisms (SNPs) and categorized these individuals into the Fujian-bred and the Crimo populations according to phylogenetic analyses. We then delved deeper into their evolutionary relationships through assessing population structure, calculating fixation index (*F*_ST_) values, and measuring genetic distances. Our exploration of runs of homozygosity (ROHs) and homozygous-by-descent (HBD) uncovered genomic regions enriched for genes implicated in fatty acid metabolism, development, and immunity pathways. Selective sweep analyses further indicated strong selective pressures exerted on genes including *TECR*, *STAT2*, and *TRAF5*. These findings provide insights into genetic variations of Muscovy ducks, thus offering valuable information regarding genetic diversity, population conservation, and genome associated with the breeding of Muscovy ducks.

## Introduction

The Muscovy duck (*Cairina moschata*), indigenous to the tropical climes of Central and South America, has been effectively introduced and cultivated across Africa, Asia, and Europe. Muscovy ducks are valued throughout the world for their low-calorie meat, low demand for feed quality, and less susceptibility to diseases (Chen *et al*. 2009; Gentile *et al*. 2024). Similar to other agricultural avians, Muscovy ducks have developed traits related to growth rates, egg production, and disease resistance during the breeding process (Bencharit *et al*. 2013; Talebi *et al*. 2020). According to the Quanzhou Prefecture Gazetteer (1743 edition, in Chinese), Muscovy ducks were introduced to Fujian Province (Fujian, China) via commercial activity between Europe and eastern China. The domestication process started in Fujian and formed multiple geographical populations, such as Putian (PT; Putian County, Fujian), Yongchun (YC; Yongchun County, Fujian), and Gutian (GT; Gutian County, Fujian) Muscovy duck populations (Chen and Zeng 1990). After around 250 years later, Groupe Grimaud company introduced the commercial Muscovy duck breed Crimo to China in 1996. The Crimo duck population has attracted significant commercial value due to its contribution for traits pertinent to production. It underwent further selective breeding and hybridization with the Fujian-bred Muscovy duck for better production performance. In particular, the Crimo populations (Crimo-101, Crimo-103, Crimo-Fujian, Crimo-R91, and Crimo-F11) demonstrate commercial breeding practices in comparison with the Muscovy duck populations in the Fujian Province of China (Fujian–GT, Fujian–YC, and Fujian–PT; Cheng *et al*. 2017; Zhu *et al*. 2022; Song *et al*. 2023). However, this effort has also made the genetic background of Chinese Muscovy duck population more ambiguous, which is not conducive to further breeding of Muscovy ducks (Wang *et al*. 2022; Letko *et al*. 2023).

To overcome this difficulty, we performed runs of homozygosity (ROHs) and homozygous-by-descent (HBD) analysis to resolve the genetic diversity of Muscovy ducks in China. ROH and HBD analyses are commonly employed in animal breeding to detect conserved and beneficial genomic segments that undergo homozygosity throughout the breeding program (Weng *et al*. 2021; Kostyunina *et al*. 2022). ROHs are contiguous homozygous genomic stretches that are indicative of inbreeding and are instrumental in assessing population genetic diversity, the potential for hereditary disorders, and the identification of regions undergoing strong selective pressures (Ceballos *et al*. 2018; Bertrand *et al*. 2019; Abdoli *et al*. 2023; Guo *et al*. 2023). For example, in Galliformes, ROH analysis has elucidated genes linked to phenotypic traits including limb development (*GREM1* and *MEOX2*) and immune response (*ROBO2*; Yuan *et al*. 2022), while in bovines, it has highlighted genes associated with production (*PCDHB7* and *UBE2H*) and meat quality traits (*TRNAG*, *MYOM2*, *AADAT*, and *RCAN1*; Deniskova *et al*. 2019; Macciotta *et al*. 2021; Nosrati *et al*. 2021; Liu *et al*. 2022). This approach enables us to comprehend the variations in homozygous genomic regions present in different populations throughout the breeding of Muscovy ducks.

HBD segment detection reveals genomic regions inherited from common ancestors that are indicative of reduced genetic variability. These segments can be used to trace inheritance patterns and identify genetic regions of interest for targeted breeding (Bakoev *et al*. 2021; Librado *et al*. 2021; Bergström *et al*. 2022; Druet and Gautier 2022). Such approaches have been extensively applied in agricultural animal genetics (Burkett *et al*. 2022), as demonstrated by the association of the silk feather trait (*PDSS2*) with HBD segments in chickens (Leeb *et al*. 2014) and the discovery of a frameshift mutation (*FA2H*) in cattle via HBD analysis (Jacinto *et al*. 2021). This approach aids in pinpointing the advantageous homozygous genomic regions crucial for effective breeding strategies. Utilizing a combination of ROH and HBD methodologies enables a more refined delineation of homozygous genomic fragments across diverse populations throughout various breeding programs.

Although HBD and ROH methods can screen potential selected genomic regions on a large scale, their resolution is still insufficient and they lack sensitivity to negative selection. Selective sweep can resolve these problems. The regions centering selected allele probably have relative low polymorphism because of linkage disequilibrium. Selective sweep identified these regions using comparison of data from multiple populations. This approach has been applied to elucidate genes in various agricultural animal breeding (Stephan 2019), including the genes related to the coat color and horn development in cattle (Druet *et al*. 2013), abdominal muscle growth in chickens, and down feather development in Beijing ducks (Zhou *et al*. 2018). We can conduct selective sweep analyses to identify candidate genes associated with conserved traits in breeding programs.

In this study, we focused on analyzing genome-wide Next-generation Sequencing data from 8 distinct Muscovy duck populations, encompassing totally 83 individuals from 8 populations. We identified approximately 11.24 million single nucleotide polymorphisms (SNPs) and classified the Muscovy ducks into Fujian-bred and Crimo populations. Using ROH, HBD, and selective sweep analyses, we identify multiple genomic regions under selection in commercial Muscovy ducks, with a significant focus on genes involved in fatty acid metabolism and immunity. These findings contribute to the commercial value and breeding enhancement of Muscovy ducks.

## Materials and methods

### Short-read genomic DNA data sequencing

Blood samples were collected from 83 Muscovy ducks for DNA extraction, ensuring the exclusion of closely related animals. These individuals were sourced from 8 populations, which can be classified into 3 groups: a purebred Crimo population in Fujian, 3 populations bred in Fujian (GT, PT, and YC), and 4 Crimo populations (101, 103, R91, and F11). The GT population is located in Gutian County in Fujian, the YC population is situated in Yongchun County in Fujian, and the PT population is based in Putian County.

Following the protocol outlined by the NextOmics Bioscience Genome Center (Wuhan, China), DNA extraction was performed on fresh blood samples from the Muscovy ducks using the QIAGEN Genome DNA Kit (QIAGEN, Hilden, Germany). Subsequently, a library was constructed with an insertion size of 400 bp and sequenced on the Illumina HiSeq Xten platform. This sequencing effort yielded a total of 1,673.4 GB PE150 paired-end reads covering 18–20 times the Muscovy duck genome.

### Identify SNP and principal component analysis

First, we conducted quality control on the acquired FASTQ data files, which involved scrutinizing read quality, ensuring the absence of contaminants or adapter sequences, and eliminating low-quality reads. Subsequently, we employed the BWA software to align the clean reads to the reference genome of Muscovy ducks (Jung *et al*. 2022). The reference genome spans 1.23 Gb, comprising 154 scaffolds, with a contig N50 of 40.41 Mb and BioSample ID SAMN35768738. This process yielded a set of alignment files, serving as input for variant calling (Shahzad *et al*. 2020). Following this, we conducted principal component analysis (PCA) labeling on the alignment files from different samples to identify and eliminate any outliers (Calafell *et al*. 2021). Subsequently, we utilized the Genome Analysis Toolkit (GATK4 version 4.01) for variant calling (Niaré *et al*. 2023). We then merged the genomic variant call format (gVCF) files generated from multiple samples and perform genotype extraction using GATK4. Subsequently, we employed BCFtools (version 1.31; Danecek *et al*. 2021) to identify and merge the VCF files generated from different samples. Next, we utilized IQ-TREE (version 1.6.5; Lanfear *et al*. 2020) to construct an evolutionary tree with 1,000 repetitions of testing. IQ-TREE is a rapid and precise tool for phylogenetic analysis based on maximum likelihood. Finally, we used the PLINK (version v1.90b6.21; Purcell *et al*. 2007) software to conduct PCA analysis on the merged data set (Gu *et al*. 2023).

### Admixture and genetic distance

Initially, we utilized the BCFtools (version 1.31) software to merge the SNP file generated in the preceding step. Subsequently, we employed VCFtools (version 0.1.17; Danecek *et al*. 2011) to filter the file based on minQ=30 (Quinlan and Hall 2010; Guo *et al*. 2021). Following this, we utilized PLINK (version v1.90b6.21) for analysis (--noweb --file PLINK _Out --hwe 0.0001 --make-bed --out QC --allow-extra-chr --chr-set 82 --geno). Finally, a population structure analysis was conducted using admixture (version 1.3.0; Yamaguchi-Kabata *et al*. 2008). The admixture results were visualized using the R language, and genetic distance analysis on the population was conducted with parameters “--west-fst-pop id4 --west-fst-pop id --fst-window-size 20000 --fst-window-step 5000” (Lipson *et al*. 2022).

### ROH detection

The PLINK software (version v1.90b6.21) was utilized to identify ROH on the autosomes (Fieder *et al*. 2021). The following criteria were employed to define ROH: (1) The homozyg-group parameter was added to obtain ROH information for the group. (2) The minimal number of SNPs in an ROH was set to 40. (3) The maximal gap between adjacent SNPs was set to 1 Mb. (4) The minimum SNP density per ROH was set to 1 SNP every 100 kb. (5) No heterozygotes were allowed in regions less than 16 Mb (assuming a genotype error rate of 0.2%). (6) Based on ROH length, the number of missing genotypes was defined as follows: ROH > 1 Mb, 0 missing genotypes; 1–2 Mb, 2 missing genotypes; 2–3 Mb; and ROH > 3 Mb. (7) The minimum length required for an ROH was set to 1 Mb.

The detected ROHs were categorized based on length into 4 categories: category 1, ROHs between 0 and 1 Mb; category 2, ROHs between 1 and 2 Mb; category 3, ROHs between 2 and 3 Mb; and category 4, ROHs above 3 Mb.

### HBD detection

HBD segments were estimated using the R package RZooRoH (v.0.3.0.74; Liao *et al*. 2021). This package is based on the hidden Markov model, which identifies HBD segments and non-HBD segments. PLINK (version v1.90b6.21) was employed to convert the generated intermediate file format. RZooRoH enables the determination of approximate generation classes based on the length of the segments. The different HBD classes are defined by their specific rates (*R*_k_). HBD segments were classified according to *R*_k_ rate series: 16, 64, 256, 512, 4,096, and 6,000, which correspond to HBD segments with ages approximately 6, 24, 96, 192, 1,536, and 2,250 years ago, respectively. The length of HBD segments from class k follows an exponential distribution with the rate *R*_k_ and mean 1/*R*_k_. Classes with lower rates correspond to longer HBD segments from more recent common ancestors. The rate of the class is approximately equal to the length of the inbreeding loop in generations. The ANNOVAR software was used for variant site detection, and the PROVEAN software was utilized for harmful mutation evaluation.

### Selective sweep

Selective sweep is a phenomenon where the diversity of multiple genes decreases through linkage or haplotype due to the selection of a certain gene (Druet *et al*. 2013). This study used a joint analysis method of pi, Tajima's *D*, SweeD (version 4.0.0; Pavlidis *et al*. 2013), and XP-CLR (version 1.1.2; Chen *et al*. 2010) to identify selective sweep regions. In this study, SweeD and XP-CLR software were used for selective sweep analysis. SweeD worked with parameters “-grid {chromosome length/50,000 window size} -minsnps 200 -maf 0.05 -missing 0.1.” We implemented selective sweep analysis by XP-CLR with parameters “--maxsnps 600 --size 50,000 --step 25,000.” The top 1 region for each chromosome was selected from the output of SweeD. As for XP-CLR, the top 0.2% of XP-CLR scores were screened as top regions in XP-CLR analysis (at least 1 region remained). In addition, this study utilized VCFtools (version 0.1.17) to calculate the values of pi and Tajima's *D*, with data sources based on variation files from ROH detection steps.

## Results

### The construction of a phylogenetic tree elucidates the relationships among the Muscovy duck populations

In this study, we sequenced 83 Muscovy individuals from 8 populations, including 1 purebred Crimo population (Fujian), 3 geographical populations from Fujian-bred (GT, PT, and YC), and 4 suspected Crimo populations (101, 103, R91, and F11) that were further domesticated in China. These data enabled us to detect SNPs and delineate the population structure (Supplementary Table 1).

**Fig. 1.**
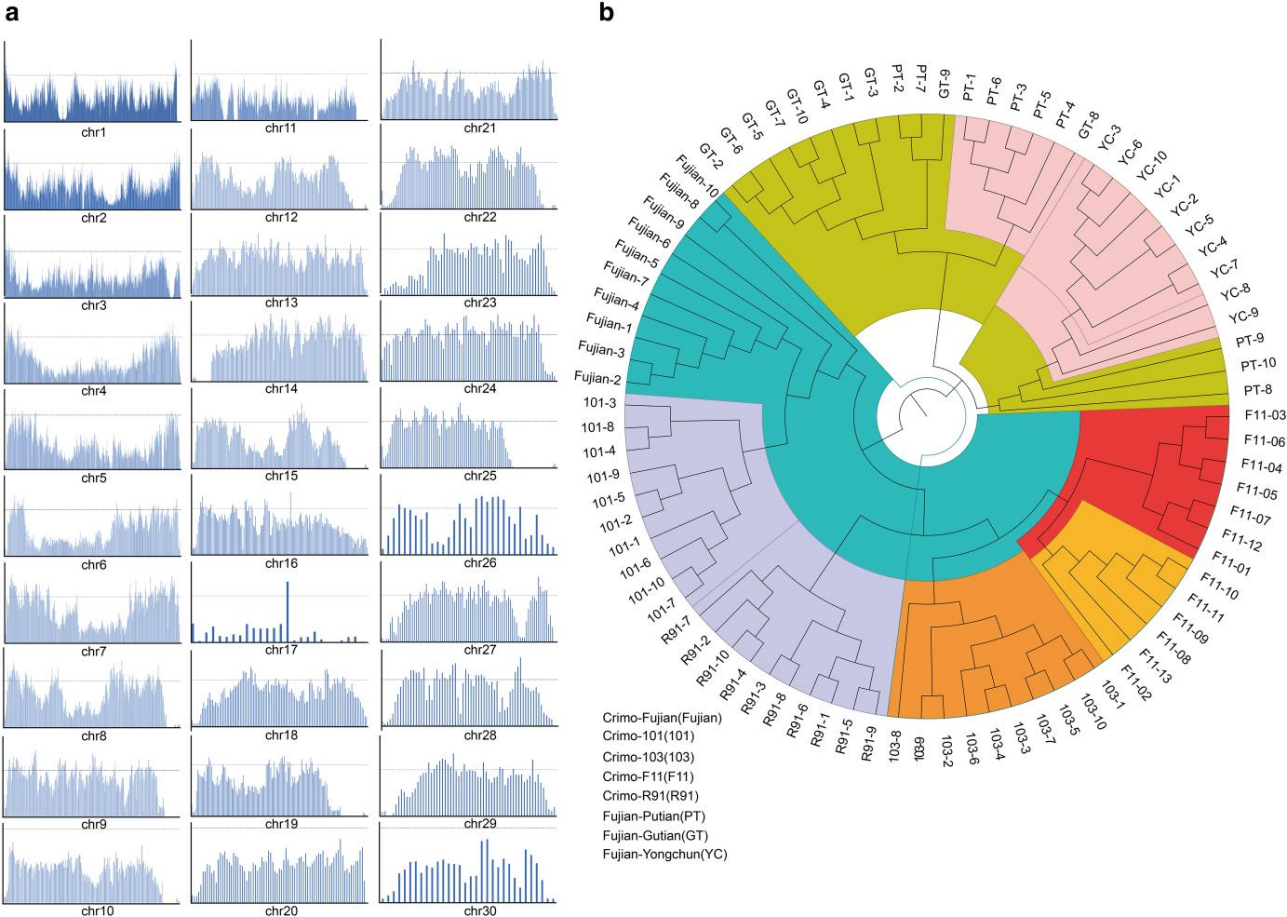
Genome-wide SNP statistics and the phylogenetic tree. a) The distribution of SNP numbers in 500 kb window on chromosomes. b) The phylogenetic tree of 83 Muscovy duck individuals.

We identified 11.24 million high-quality SNPs, among which 0.73 million were situated within gene regions, and 0.16 million were found within exon regions. Most variations were located in noncoding sequences, including intergenic and intronic regions, indicating that noncoding sequences that have the potential to change protein function by regulating gene expression were retained during evolution and domestication. Our analysis highlighted a notable variation in SNP density across the genome. To be specific, a higher SNP density was observed near the telomere regions of macrochromosomes (chr1-9) when analyzed with the use of a 100-kb sliding window. By contrast, a lower density was recorded in the microchromosomes (chr10-16 and chr18-28) and dot chromosomes (chr17 and chr29-30; Fig. 1a; Huang *et al*. 2023).

We then constructed a phylogenetic tree based on the SNP data, which can further reveal the evolutionary relationships of these 8 Muscovy duck populations (Fig. 1b). The 103, 101, Fujian, R91, and F11 populations formed the Crimo Muscovy duck population clade. The GT, YC, and PT populations formed the Fujian-bred clade. According to this information, we classified the 8 population into 2 main classes, including Crimo (Crimo–Fujian, Crimo-101, Crimo-103, Crimo-F11, and Crimo-R91) and Fujian-bred (Fujian–GT, Fujian–PT, and Fujian–YC) classes.

### Population structure elucidates the classification of 8 Muscovy duck populations

**Fig. 2.**
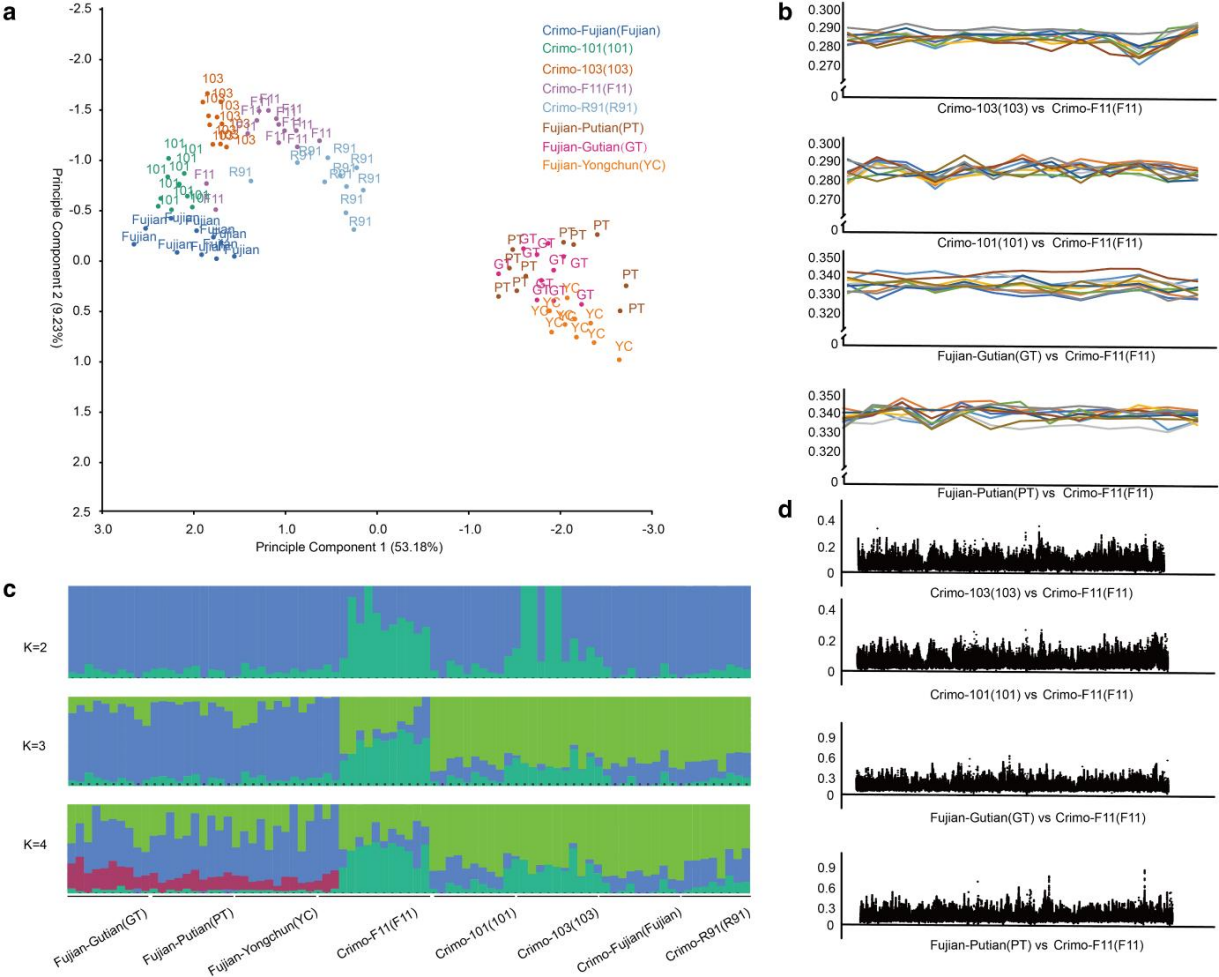
Analysis of the population structure. a) Scatter plots of PCA for 8 Muscovy duck populations. b) The admixture analysis of population structure differences among 83 individuals. c) The comparison of genetic distances between Fujian-bred and Crimo populations. d) The comparison of *F*_ST_ between Fujian-bred and Crimo populations.

We analyzed the genetic structure of Muscovy duck populations, which could facilitate our understanding of their genetic diversity. The relationships among individuals were identified using PCA. The first component (PC1) and the second component (PC2) exhibit a separation between the commercial Crimo Muscovy duck populations (Crimo-101, Crimo-103, Crimo-F11, Crimo-R91, and Crimo–Fujian populations) and the Fujian-bred populations (Fujian–PT, Fujian–GT, and Fujian–YC populations; Fig. 2a).

We performed a clustering approach implemented in ADMIXTURE to identify the population structure of these Muscovy ducks. When determining *K* values through cross-validation, the optimal *K* value is chosen as the value minimizing the cross-validation error. At *K* = 3, the populations in Fujian-bred class were clearly separated from the commercial Crimo populations (Fig. 2b; Supplementary Fig. 1).

Besides, we also estimated the genetic distances within and between these populations. The genetic distances between individuals from different class were higher than those observed within the same class. For example, the genetic distance between individuals from the 103 and F11 populations ranged from 0.280 to 0.290, while the distance between PT and F11 varied from 0.330 to 0.350 (Fig. 2c).

Our fixation index (*F*_ST_) analysis compared the genetic variance between the Crimo and Fujian populations. The *F*_ST_ values calculated by comparison between populations from the same class ranged from 0 to 0.4, while those by comparison between populations from the different class ranged from 0 to 0.6 (Fig. 2d).

### ROH analysis reveals homozygous regions overlapping immunity and fatty acid metabolism genes

**Fig. 3.**
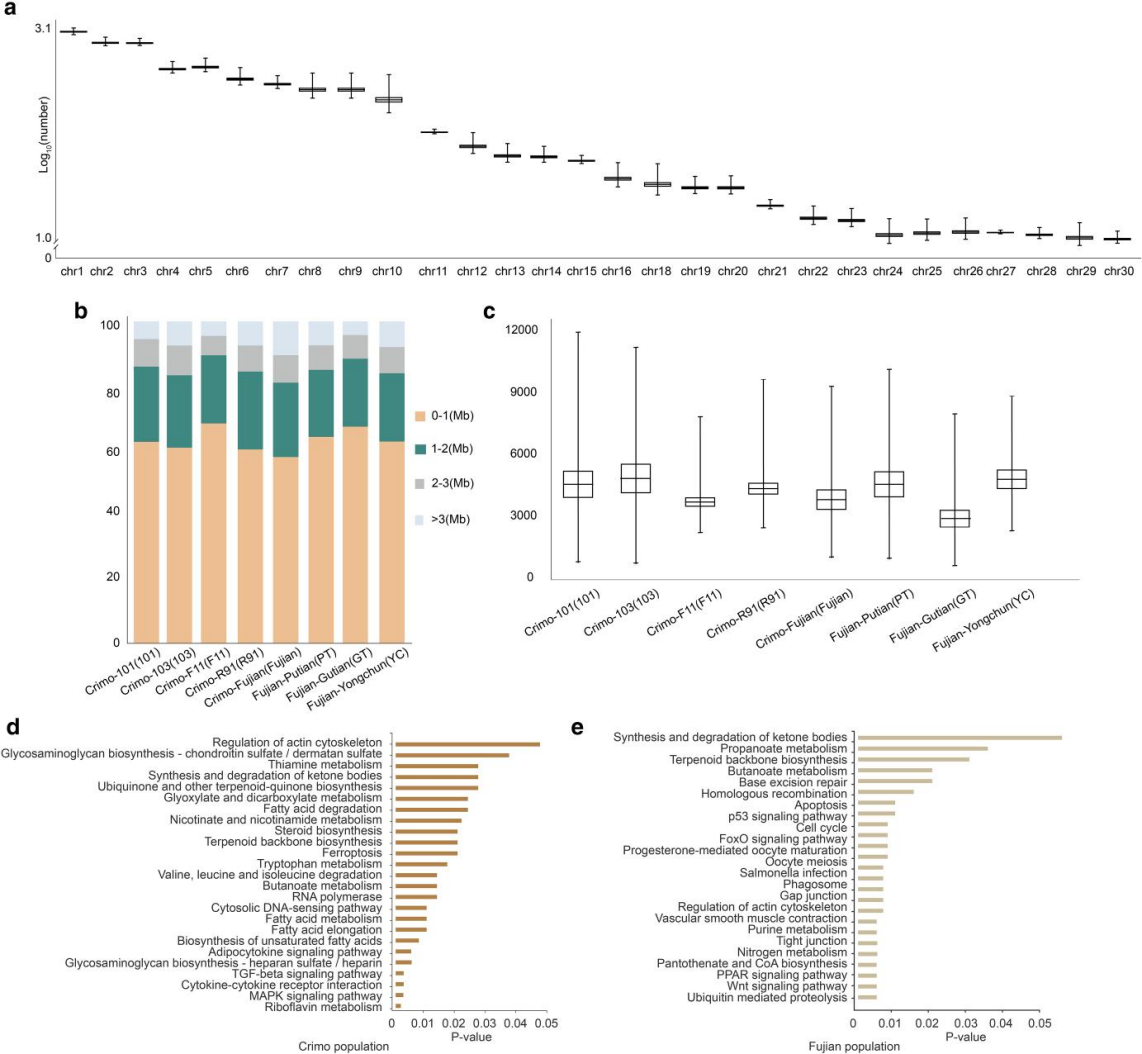
Statistics of ROH and KEGG enrichment analysis. a) The number (log_10_) of ROH fragments in the whole genome. b) The proportion of different ROH fragment length. (c) The length statistics of ROH in 8 populations. d) The results of KEGG enrichment analysis in commercial population ROH-overlapping genes. e) The results of KEGG enrichment analysis in Fujian-bred population ROH-overlapping genes.

To determine selection regions, we implemented the ROH analysis based on the data from these 83 Muscovy ducks. Our investigation showed an average of 5,681 ROHs per individual (Fig. 3a). When categorizing these ROHs by their lengths, we found that approximately 60, 25, 10, and 5% were 0–1, 1–2, 2–3, and >3 Mb in length, respectively (Fig. 3b). The overall mean length of ROHs across all individuals was 1,138,123 bp, while among the populations studied, population 101 stood out with the highest length of ROHs being 12,984,131 bp (Fig. 3c). ROH islands might be indicative of genomic regions that underwent natural and/or artificial selection. We identified 55–72 regions of the genome with a high frequency of ROH occurrence, also known as ROH islands (Supplementary Fig. 2). Within 48 ROH islands, we identified 463 genes for all populations.

To further our understanding of the potential functional implications of these genes with overlapped ROHs, we performed a Kyoto Encyclopedia of Genes and Genomes (KEGG) enrichment analysis on genes located within ROH regions (Fig. 3d and e). Additionally, we performed a Gene Ontology (GO) enrichment analysis on these genes between commercial populations and Fujian-bred populations (Supplementary Figs. 3 and 4).

GO and KEGG analyses showed that these genes are enriched in 67 terms, some of which were related to fatty acid metabolism, immunity, and development. The KEGG terms included fatty acid degradation, MAPK signaling pathway, and regulation of actin cytoskeleton. In the GO terms, the most included phosphatidylinositol-4,5-bisphosphate binding, tyrosine phosphorylation of STAT protein, and growth factor activity. Intriguingly, such genes were absent in the ROH-overlapping genes of the Fujian-bred populations. This also highlights a distinct genetic basis for variations in fatty acid metabolism, development, and immunity pathways between the Fujian-bred and commercial Crimo populations.

### The detection of HBD indicated homozygous regions related to immunity and fatty acid metabolism genes

**Fig. 4.**
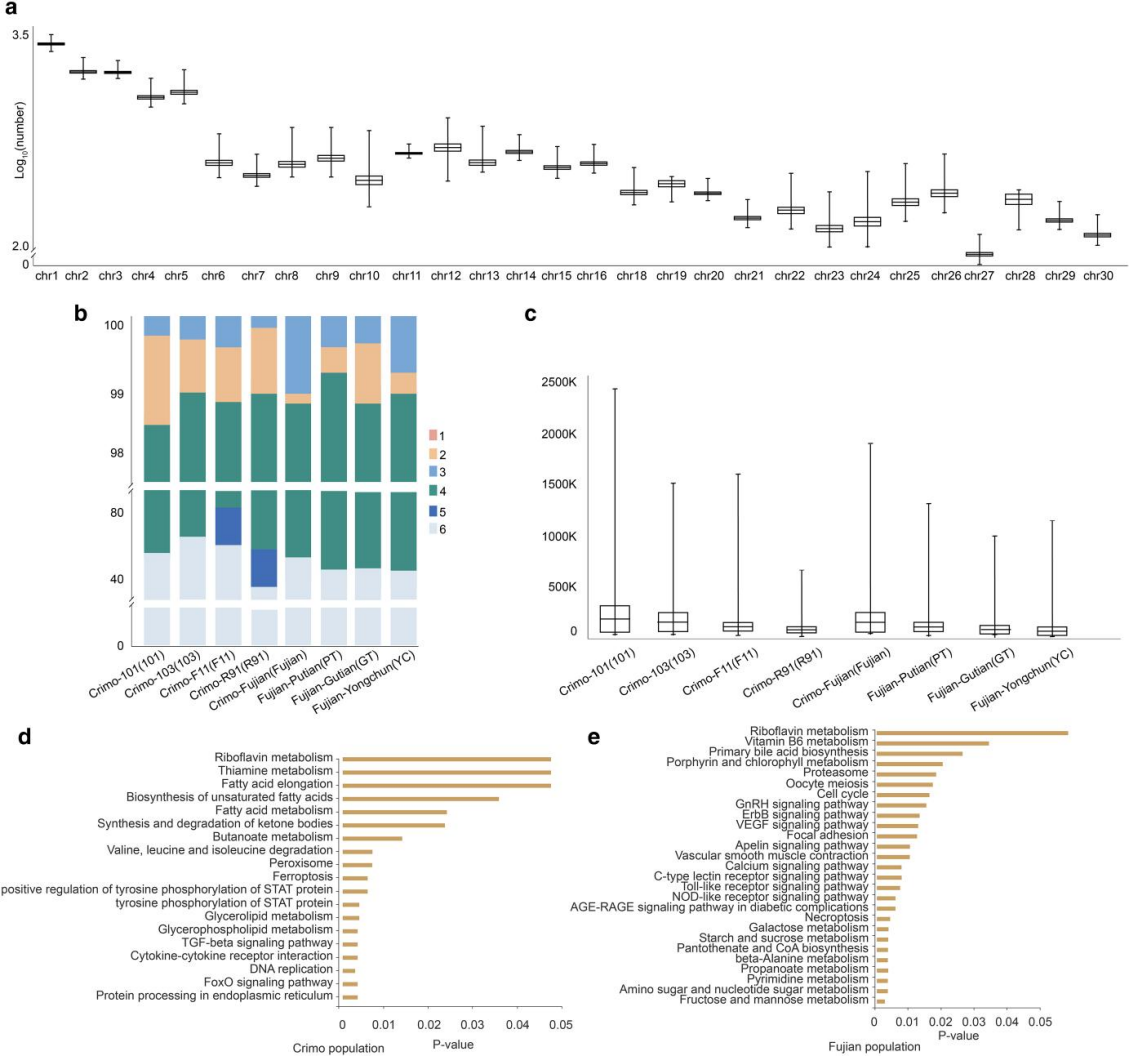
Statistics of HBD and KEGG enrichment analysis. a) The number (log_10_) of HBD fragments in the entire genome. b) The proportion of HBD at different levels. c) The length statistics of HBD in 8 populations. d) The results of KEGG enrichment analysis in the commercial populations in HBD-overlapping genes. e) The results of KEGG enrichment analysis in the Fujian-bred populations in HBD-overlapping genes.

To further identify the breeding hotspot and estimate the ages of selective regions, we performed HBD analysis to classify the homozygous regions into different history time slots. Our findings indicated that the number of HBD segments spans from 24,398 to 32,653 across the Muscovy duck genome (Fig. 4a). These segments represent a spectrum of genetic homozygosity, with levels 4–6 denoting fragments homozygous during ancient times and levels 1–3 corresponding to more recent domestication efforts. HBD fragments at levels 1, 2, and 3 are reflective of homozygosity events occurring within the past 6, 24, and 100 years, respectively. Our analysis across various populations indicated that modern homozygous fragments (levels 1–3) constitute less than 3% of the total, while ancient homozygous fragments (levels 4–6) occupy approximately 97% (Fig. 4b). The largest HBD fragment identified was 2,400 kb, with an average length of 110.735 kb for all segments (Fig. 4c).

We conducted a comparative analysis of the genomic regions delineated by the ROH fragment and the HBD fragment between Crimo and Fujian populations. Crimo class had 22,173 ROH fragments and HBD segments overlapping regions, including 373 genes that were unique to the Crimo populations, associating with fatty acid metabolism, immunity, and development according to KEGG and GO enrichment analyses. The KEGG terms included biosynthesis of unsaturated fatty acids, tyrosine phosphorylation of STAT protein, and fatty acid elongation. In the GO terms, the most included phosphatidylinositol-4,5-bisphosphate binding, tyrosine phosphorylation of STAT protein, and growth factor activity. These analyses further ensured that genes within selection regions of Crimo populations were enriched for roles in the fatty acid metabolism, development, and immunity pathways, distinguishing them from the ones observed in the Fujian-bred populations (Supplementary Figs. 5 and 6 and Tables 2 and 3).

We subsequently assessed nonsynonymous mutations in these 373 genes using the PROVEAN software, which assigned a deleterious mutation score < −2.5. Notably, mutations in the exon regions of genes such as *HACD1*, *TECR*, *TRAF5*, and *IL21* were identified with deleterious scores of −2.8, −2.6, −2.9, and −2.9, underscoring their potential adverse effects on populations in the Fujian class (Supplementary Table 4).

In summary, ROH and HBD analyses uncovered selective genomic regions overlapping genes related to the fatty acid metabolism, development, and immunity pathways. The identified genes *HACD1*, *TECR*, *TRAF5*, and *IL21* and corresponding mutations highlight the influence of breeding process on traits related to growth, immunity, and meat quality of Muscovy ducks.

### Selective sweep unveils selection pressures on 122 genes

While ROH and HBD analyses can identify as many selective regions as possible, these methods have insufficient resolution and are not sensitive to negative selection. Thus, we performed selective sweep based on the data from Fujian-bred and Crimo populations. Crimo Muscovy duck populations showed numerous superior traits during domestication, including high growth rate, heavier body weight, and lower meat fat, which are different from the other noncommercial populations. We implemented selective sweep analysis in the Crimo Muscovy duck populations and Fujian-bred populations. Through the comparisons of these Muscovy duck populations by the SweeD software, we identified a total of 42,686 genomic regions under selection (Supplementary Table 5).

**Fig. 5.**
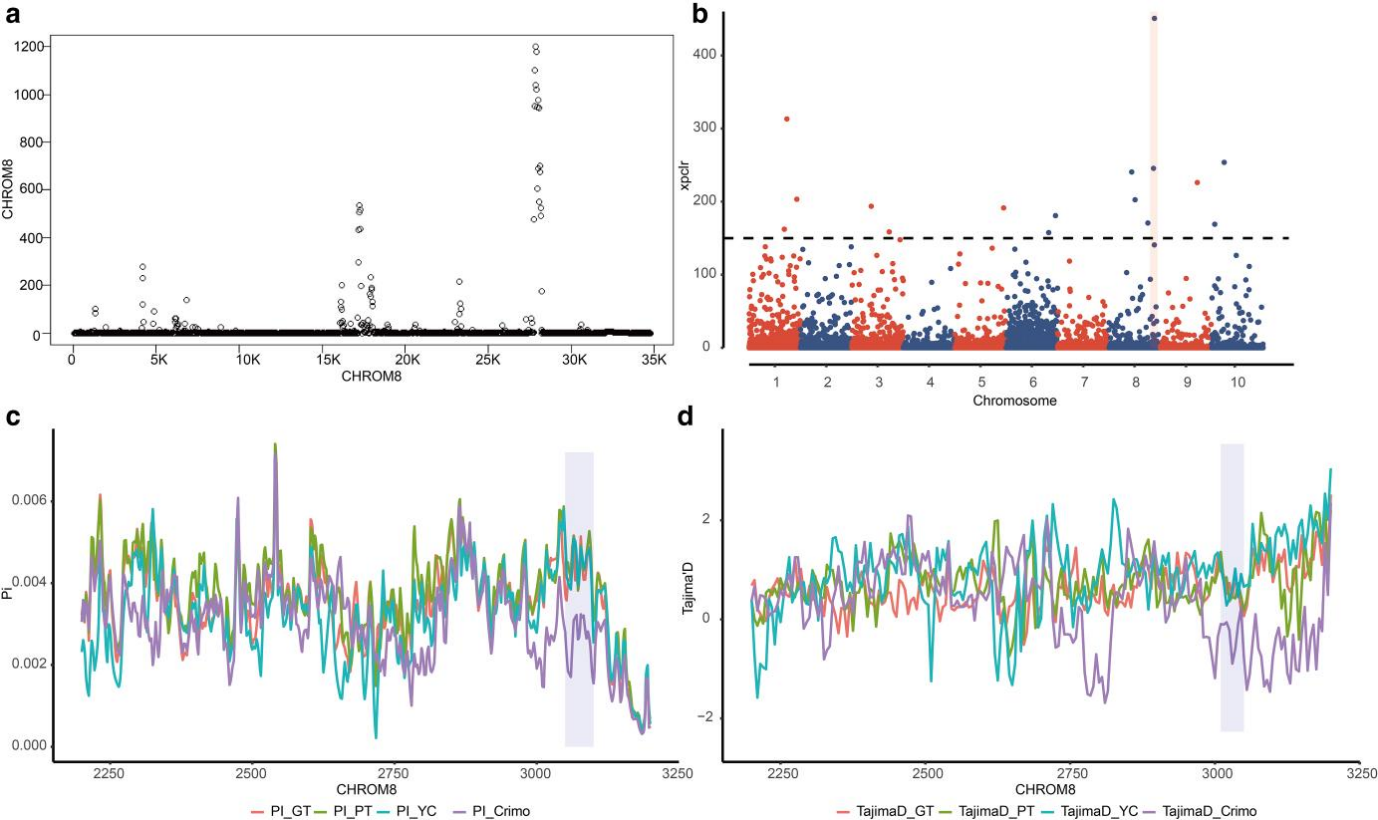
Selective sweep unveils selection pressures on *TECR*. a) The identification of selective regions on chromosome 8 by SweeD. b) XP-CLR value distribution on chromosomes 1–10. c) The pi analysis results of Fujian-bred and commercial populations on chromosome 8. d) The Tajima's *D* analysis results of Fujian-bred and commercial populations on chromosome 8.

Among the 42,686 genomic regions detected by SweeD, we screened out regions with the highest likelihood from 38 chromosomes, totally containing 3,286 genes. We further narrowed the selective region through XP-CLR value analysis and identified 62 regions containing 122 genes. Among the intersection between SweeD- and XP-CLR-identified regions, chr8: 30,500,182–30,624,822 contains the *TECR* gene, which functions in the last synthesis step of long-chain fatty acids and may result in a higher sebum rate in Muscovy ducks (Fig. 5a and b).

We further calculated the pi (regional nucleic acid polymorphism) and Tajima's *D* in region chr8: 30,500,182–30,624,822 (Fig. 5c). We found Tajima's *D* < 0 in Crimo Muscovy duck population, while Tajima's *D* > 0 was observed in this region of the 3 populations of Fujian-bred Muscovy duck (Fig. 5d). Among the 3 sliding windows of pi in chr8: 30,500,182–30,624,822, the pi of French Crimo Muscovy duck populations were found to be lower than that of the other 3 Fujian-bred Muscovy duck populations, with significance values of 0.0017, 0.0024, and 0.0025 compared with PT, GT, and YC Muscovy ducks, respectively. These results demonstrate that chr8: 30,500,182–30,624,822 was under selection in the Crimo Muscovy duck, and artificial selection may exert an influence on its *TECR* function. This may contribute to the breeding of low-fat meat Muscovy ducks in addition to the *TECR* gene associated with fatty synthesis. We also found 2 immune-related genes in other genomic regions under selection, *STAT2* and *TRAF5*, which are involved in diseases such as herpes simplex virus 1 and influenza A infection.

## Discussion

In our study, we identified 11.24 million high-quality SNPs derived from both Fujian-bred and commercial Crimo populations. Subsequently, ROH, HBD, and selective sweep analyses were conducted to identify genes related to immunity and fatty acid metabolism among the populations. In addition, the insights garnered from this endeavor have significantly advanced our understanding of the genetic underpinnings associated with these critical traits in Muscovy ducks.

In agricultural meat products, the relationship between fat content and meat texture and taste is shown to be complex and multifaceted (Boz *et al*. 2019). As an economically important poultry breed, Muscovy ducks are now farmed worldwide for meat consumption due to their leanness, tenderness, and taste and are an essential source of income in many rural communities, especially in developing countries in Africa (Zeng *et al*. 2015; Xu *et al*. 2022). The detailed analysis of Fujian-bred and Crimo populations unveiled specific homozygous regions harboring genes related to fatty acid metabolism (*HACD1*, *ELOVL4*, *SCD5*, and *TECR*; Fieder *et al*. 2021; Supplementary Tables 2 and 3). These fatty acid metabolism genes probably have influence on the fat content in meat and further change the meat taste of Muscovy duck. We found a G to A nonsynonymous mutation in the exon of *HACD1* of the commercial populations compared with Fujian-bred Muscovy duck populations. Moreover, our findings revealed a critical nonsynonymous mutation in the *HACD1* gene, distinguishing the commercial from the Fujian-bred Muscovy duck populations. In the production of pork, muscle mass can be adjusted by adjusting *HACD1* (Chen *et al*. 2019). In mice, *ELOVL4* are responsible for the biosynthesis of very-long-chain saturated and polyunsaturated fatty acids (Hopiavuori *et al*. 2019). In Meishan pigs, MSTN can act through the *SCD5* to regulate fatty acid metabolism (Ren *et al*. 2020). These alterations in the coding protein may contribute to the difference from the Fujian-bred populations of the fatty acid metabolism ability in the commercial populations. The genes associated with fatty acid metabolism pathways exhibit deleterious mutations that could make potential impact on the fatty acid metabolism of the Fujian-bred populations. By investigating and manipulating these genes during breeding, it may become feasible to modify the fatty acid metabolism of Muscovy ducks, aiming to enhance their flavor profile for the local market demand (Wood *et al*. 2008).

In addition to fatty acid metabolism in Muscovy ducks, disease resistance ability was also of great importance. Immune competence in Muscovy ducks is of particular importance for backyard farmers who lack specialized rearing experience and facilities. Our study highlighted the genes including *IL21* and *KIT*, which play crucial roles in activating T cells and modulating the immune response (Supplementary Tables 2 and 3; Nash *et al*. 2022). The differential expression of these genes in response to environmental factors, including rearing systems, underscores the interplay between genetics and external conditions in shaping immune capabilities. In chicken research, the rearing system exerted significant influence on the jejunum expression of *IL-10*, *IL-2*, and *IL-6*, where these genes were upregulated in a free-range system (Stefanetti *et al*. 2023). A significant interaction between the rearing system and the genotype was also shown. More specifically, native breeds exhibited a significantly higher expression (*P* < 0.001) of *IL-6* in the free-range system compared with the same genotypes in the conventional system (Stefanetti *et al.* 2023). We identified nonsynonymous mutations in the exon regions of *KIT*, *IL21*, and *TRAF5* genes between Fujian-bred and Crimo populations (Supplementary Table 4). Both the *TRAF5* and *IL21* genes in Fujian-bred populations harbor deleterious mutations. This may be caused by different feeding environments, where poultry is exposed to pathogen, which could thereby promote immune development (Jeni *et al*. 2021). This variant information can serve as a reference for the breeding of Fujian-bred populations, with the purpose of improving their immune capabilities and subsequently increasing production.

In conclusion, our investigation into the genetic diversity of Muscovy ducks has illuminated genetic factors influencing fatty acid metabolism and immunity. Through identifying genetic hotspots within the Crimo Muscovy duck populations, we have established a theoretical foundation for developing breeding strategies. These strategies aim to produce Muscovy ducks with optimized fat content and enhanced disease resistance, ultimately elevating the commercial value of this species.

## Supplementary Material

jkae112_Supplementary_Data

## Data Availability

The data underlying this article are available in the NCBI and can be accessed with BioProject: PRJNA1019115 and PRJNA984447. The script can be obtained from the figshare at https://doi.org/10.6084/m9.figshare.24912501.v2. Supplemental material available at G3 online.
