# Supplementary material for: A comparative analysis reveals the genomic diversity among 8 Muscovy duck populations
Source: G3 (Bethesda). 2024 May 24;14(7):jkae112. doi: 10.1093/g3journal/jkae112 (PMC11228869; doi:10.1093/g3journal/jkae112)
Supplement: jkae112_Supplementary_Data [file jkae112_supplementary_data.docx]

**Supplementary Tables**

**Table S1** Statistics of sequencing in 83 individuals

| Sample | Size | Sequencing Depth | Breed | Mapping rate |
| --- | --- | --- | --- | --- |
| 0141A | 18.9 GB | 16 | Crimo-101 | 98.11 |
| 0147A | 18.2 GB | 15 | Crimo-101 | 97.17 |
| 0157A | 18.5 GB | 15 | Crimo-101 | 98.02 |
| 0195A | 18.5 GB | 15 | Crimo-101 | 97.66 |
| 0606A | 18.8 GB | 16 | Crimo-101 | 96.63 |
| 0610A | 18.5 GB | 15 | Crimo-101 | 96.32 |
| 0647A | 18.3 GB | 15 | Crimo-101 | 97.13 |
| 0688A | 18.6 GB | 15 | Crimo-101 | 96.66 |
| 0699A | 18.5 GB | 15 | Crimo-101 | 97.22 |
| 0700A | 18.9 GB | 16 | Crimo-101 | 96.33 |
| 7024A | 18.3 GB | 15 | Crimo-103 | 95.52 |
| 7568A | 18.7 GB | 16 | Crimo-103 | 96.31 |
| 7618A | 18.4 GB | 15 | Crimo-103 | 96.11 |
| 8424A | 18.6 GB | 15 | Crimo-103 | 96.82 |
| 8697A | 18.5 GB | 15 | Crimo-103 | 96.37 |
| 8702A | 18.6 GB | 16 | Crimo-103 | 95.28 |
| 8866A | 18.8 GB | 16 | Crimo-103 | 94.99 |
| 9151A | 18.6 GB | 15 | Crimo-103 | 95.88 |
| 9316A | 18.2 GB | 15 | Crimo-103 | 95.02 |
| 9318A | 18.3 GB | 15 | Crimo-103 | 95.63 |
| B1901A | 20.9 GB | 17 | Putian | 98.02 |
| B1914A | 20.8 GB | 17 | Putian | 98.10 |
| B1961A | 20.5 GB | 17 | Putian | 97.95 |
| B1962A | 21.1 GB | 18 | Putian | 97.89 |
| B2024A | 21.0 GB | 18 | Putian | 97.66 |
| B2060A | 20.8 GB | 17 | Putian | 97.32 |
| B2094A | 21.1 GB | 18 | Putian | 98.03 |
| B2229A | 21.1 GB | 18 | Putian | 98.11 |
| B2236A | 19.0 GB | 16 | Putian | 97.32 |
| B2241A | 21.1 GB | 18 | Putian | 97.47 |
| C2072A | 20.9 GB | 17 | Crimo-Fujian | 94.21 |
| C2092A | 21.8 GB | 18 | Crimo-Fujian | 94.66 |
| C2099A | 21.3 GB | 18 | Crimo-Fujian | 94.29 |
| C2105A | 19.7 GB | 16 | Crimo-Fujian | 94.83 |
| C2128A | 21.4 GB | 18 | Crimo-Fujian | 94.18 |
| C2143A | 21.9 GB | 18 | Crimo-Fujian | 94.85 |
| C2172A | 21.9 GB | 18 | Crimo-Fujian | 94.63 |
| C2178A | 21.0 GB | 18 | Crimo-Fujian | 94.68 |
| C2186A | 21.3 GB | 18 | Crimo-Fujian | 94.63 |
| C2207A | 21.3 GB | 18 | Crimo-Fujian | 94.99 |
| FY01 | 18.5 GB | 15 | Crimo-F11 | 95.66 |
| FY02 | 18.5 GB | 15 | Crimo-F11 | 95.81 |
| FY03 | 18.3 GB | 15 | Crimo-F11 | 95.63 |
| FY04 | 17.9 GB | 15 | Crimo-F11 | 95.28 |
| FY05A | 21.7 GB | 18 | Crimo-F11 | 96.11 |
| FY06 | 18.2 GB | 15 | Crimo-F11 | 96.37 |
| FY07 | 18.8 GB | 16 | Crimo-F11 | 94.85 |
| FY08A | 20.9 GB | 17 | Crimo-F11 | 96.36 |
| FY09A | 21.6 GB | 18 | Crimo-F11 | 96.48 |
| FY10A | 20.8 GB | 17 | Crimo-F11 | 96.52 |
| FY11A | 21.6 GB | 18 | Crimo-F11 | 96.17 |
| FY12A | 20.6 GB | 17 | Crimo-F11 | 95.29 |
| FY13A | 19.6 GB | 16 | Crimo-F11 | 95.68 |
| GTBF-1A | 21.0 GB | 17 | Gutian | 98.02 |
| GTBF-2A | 20.9 GB | 17 | Gutian | 97.63 |
| GTBF-3A | 21.3 GB | 18 | Gutian | 97.66 |
| GTBF-4A | 20.9 GB | 17 | Gutian | 97.84 |
| GTBF-5A | 21.0 GB | 18 | Gutian | 97.92 |
| GTBF-6A | 20.7 GB | 17 | Gutian | 98.01 |
| GTBF-7A | 21.2 GB | 18 | Gutian | 98.00 |
| GTBF-8A | 20.5 GB | 17 | Gutian | 98.11 |
| GTBF-9A | 20.8 GB | 17 | Gutian | 97.70 |
| GTBF-10A | 20.9 GB | 17 | Gutian | 97.83 |
| R91-BF1A | 20.4 GB | 17 | Crimo-R91 | 94.99 |
| R91-BF2A | 21.1 GB | 18 | Crimo-R91 | 94.36 |
| R91-BF3A | 21.1 GB | 18 | Crimo-R91 | 94.88 |
| R91-BF4A | 20.0 GB | 17 | Crimo-R91 | 95.67 |
| R91-BF5A | 21.0 GB | 18 | Crimo-R91 | 95.23 |
| R91-BF6A | 21.2 GB | 18 | Crimo-R91 | 95.69 |
| R91-BF7A | 21.1 GB | 18 | Crimo-R91 | 95.32 |
| R91-BF8A | 20.6 GB | 17 | Crimo-R91 | 96.01 |
| R91-BF9A | 20.8 GB | 17 | Crimo-R91 | 96.77 |
| R91-BF10A | 20.6 GB | 17 | Crimo-R91 | 95.31 |
| YCBF-1A | 21.4 GB | 18 | Yongchun | 97.23 |
| YCBF-2A | 20.3 GB | 17 | Yongchun | 97.68 |
| YCBF-3A | 20.6 GB | 17 | Yongchun | 97.82 |
| YCBF-4A | 20.6 GB | 17 | Yongchun | 97.25 |
| YCBF-5A | 20.9 GB | 17 | Yongchun | 98.01 |
| YCBF-6A | 20.7 GB | 17 | Yongchun | 97.66 |
| YCBF-7A | 21.5 GB | 18 | Yongchun | 97.45 |
| YCBF-8A | 20.6 GB | 17 | Yongchun | 97.16 |
| YCBF-9A | 21.3 GB | 18 | Yongchun | 97.08 |
| YCBF-10A | 20.8 GB | 17 | Yongchun | 97.09 |

**Table S2** List of genes implicated in differential enrichment analysis of gene ontology pathways.

| Term | ID | Input |
| --- | --- | --- |
| positive regulation of tyrosine phosphorylation of STAT protein | GO:0042531 | IL21\|FYN\|IL15\|KIT |
| tyrosine phosphorylation of STAT protein | GO:0007260 | IL21\|IL15 |
| very long-chain fatty acid biosynthetic process | GO:0042761 | HACD1\|ELOVL4 |
| fatty acid elongation | GO:0030497 | HACD1 |
| unsaturated fatty acid biosynthetic process | GO:0006636 | ELOVL4\|SCD5 |
| cytokine activity | GO:0005125 | IL21\|BMP3\|LITAF\|IL15\|IL6\|SPP1 |

**Table S3** List of genes implicated in differential enrichment analysis of KEGG pathways.

| Term | Database | Input |
| --- | --- | --- |
| MAPK signaling pathway | KEGG | AREG\|PDGFRA\|RAC1\|CACNB2\|KIT\|MAPK10\|EREG\|FGF5\|FGF2\|KDR |
| Fatty acid metabolism | KEGG | HACD1\|ELOVL4\|SCD5 |
| Cytokine-cytokine receptor interaction | KEGG | IL21\|BMP3\|IL15\|IL-2\|IL6\|IL8 |
| AGE-RAGE signaling pathway in diabetic complications | KEGG | IL6\|MAPK10\|IL8\|RAC1 |
| Fatty acid elongation | KEGG | HACD1\|ELOVL4 |
| NOD-like receptor signaling pathway | KEGG | IL6\|MAPK10\|IL8\|ANTXR2 |
| Cytosolic DNA-sensing pathway | KEGG | IL6\|POLR3E |
| FoxO signaling pathway | KEGG | IL6\|MAPK10\|PLK4\|SETD7 |
| C-type lectin receptor signaling pathway | KEGG | IL6\|MAPK10\|IL-2 |
| N-Glycan biosynthesis | KEGG | SRD5A3\|MGAT4D |
| Cellular senescence | KEGG | IL6\|LIN54\|CCNA2\|IL8 |
| Influenza A | KEGG | IL6\|TMPRSS2\|IL8 |
| Melanogenesis | KEGG | KIT |

**Table S4** Mutation site detection and deleterious mutation evaluation

| gene | Term | site | Crimo populations | Fujian populations | **Prediction** |
| --- | --- | --- | --- | --- | --- |
| ELOVL4 | synonymous | chr3 34546304 | A | G | # |
| ELOVL4 | nonsynonymous | chr3 34546365 | G | A | 1.3 |
| HACD1 | nonsynonymous | chr2 136460953 | A | G | 1.5 |
| HACD1 | nonsynonymous | chr2 136467313 | C | T | -2.8 |
| IL21 | synonymous | chr4 33671074 | C | T | # |
| IL21 | synonymous | chr4 33671591 | A | G | # |
| IL21 | synonymous | chr4 33724310 | T | C | # |
| IL21 | nonsynonymous | chr4 33665302 | T | A | 1.5 |
| IL21 | nonsynonymous | chr4 33671574 | G | T | -2.9 |
| KIT | synonymous | chr1 49657302 | C | T | # |
| KIT | nonsynonymous | chr1 49674613 | C | T | 1.4 |
| SCD5 | nonsynonymous | chr4 32216168 | G | A | 1.3 |
| SCD5 | nonsynonymous | chr4 32216216 | T | C | -2.1 |
| TECR | synonymous | chr4 62650425 | A | G | # |
| TECR | synonymous | chr4 62683546 | G | A | # |
| TECR | synonymous | chr4 62696903 | T | C | # |
| TECR | nonsynonymous | chr4 62705211 | C | T | 1.1 |
| TECR | nonsynonymous | chr4 62706801 | T | C | -2.6 |
| TRAF5 | synonymous | chr3 95944781 | C | T | # |
| TRAF5 | synonymous | chr3 95948233 | G | A | # |
| TRAF5 | synonymous | chr3 95948374 | C | T | # |
| TRAF5 | synonymous | chr3 95948449 | G | T | # |
| TRAF5 | synonymous | chr3 95948536 | G | A | # |
| TRAF5 | synonymous | chr3 95948569 | A | G | # |
| TRAF5 | nonsynonymous | chr3 95945262 | G | A | -2.9 |
| TRAF5 | nonsynonymous | chr3 95945713 | C | G | -3.1 |

**Table S5** Statistics of Selective Sweep areas

| chrom | pos_start | pos_stop | xpclr | xpclr_norm |
| --- | --- | --- | --- | --- |
| chr1 | 2750131 | 2799964 | 313.0981 | 36.05067 |
| chr1 | 50400227 | 50449566 | 203.223 | 23.347 |
| chr1 | 50225045 | 50274776 | 162.2266 | 18.60702 |
| chr1 | 50675090 | 50724916 | 138.2329 | 15.83288 |
| chr1 | 50075479 | 50124875 | 125.6676 | 14.38009 |
| chr1 | 50800374 | 50849410 | 125.6128 | 14.37376 |
| chr1 | 1.2E+08 | 1.2E+08 | 123.8873 | 14.17426 |
| chr1 | 50350045 | 50399786 | 121.8093 | 13.934 |
| chr1 | 50975203 | 51024896 | 121.7216 | 13.92386 |
| chr1 | 50100236 | 50149855 | 121.3002 | 13.87513 |
| chr1 | 2350003 | 2399905 | 118.8046 | 13.5866 |
| chr1 | 50275203 | 50324771 | 112.2981 | 12.83432 |
| chr1 | 51025466 | 51074342 | 110.2419 | 12.59658 |
| chr1 | 37750046 | 37799549 | 109.6594 | 12.52924 |
| chr1 | 50525392 | 50574860 | 105.2406 | 12.01833 |
| chr1 | 50650029 | 50699645 | 102.0952 | 11.65467 |
| chr1 | 1.18E+08 | 1.18E+08 | 100.5429 | 11.47519 |
| chr1 | 85725045 | 85774920 | 100.2988 | 11.44697 |
| chr1 | 50725085 | 50774262 | 100.2282 | 11.43881 |
| chr2 | 47825079 | 47874993 | 138.1361 | 23.50769 |
| chr2 | 4750130 | 4799959 | 134.6127 | 22.90463 |
| chr2 | 4900019 | 4949974 | 116.2852 | 19.76773 |
| chr2 | 47900361 | 47949942 | 113.8166 | 19.34522 |
| chr2 | 4700636 | 4749983 | 112.4752 | 19.11563 |
| chr2 | 4650016 | 4699785 | 97.76505 | 16.59786 |
| chr2 | 38000179 | 38049932 | 94.63507 | 16.06214 |
| chr2 | 47000769 | 47049795 | 73.19989 | 12.39333 |
| chr2 | 37250127 | 37299812 | 72.82305 | 12.32883 |
| chr2 | 47175116 | 47224911 | 72.53254 | 12.27911 |
| chr2 | 4850099 | 4899888 | 71.47047 | 12.09733 |
| chr2 | 34750199 | 34799971 | 70.90585 | 12.00069 |
| chr2 | 1.14E+08 | 1.14E+08 | 69.96187 | 11.83912 |
| chr2 | 38025289 | 38074883 | 68.65295 | 11.61509 |
| chr2 | 4350050 | 4399916 | 68.63552 | 11.6121 |
| chr2 | 1.4E+08 | 1.4E+08 | 66.26652 | 11.20663 |
| chr2 | 1.4E+08 | 1.4E+08 | 63.39736 | 10.71555 |
| chr2 | 1.34E+08 | 1.34E+08 | 62.33836 | 10.53429 |
| chr2 | 1.4E+08 | 1.4E+08 | 61.96162 | 10.46981 |
| chr3 | 95925028 | 95974651 | 193.6168 | 24.00821 |
| chr3 | 95975382 | 96024945 | 158.5534 | 19.63336 |
| chr3 | 95300035 | 95349981 | 147.8104 | 18.29296 |
| chr3 | 1.03E+08 | 1.03E+08 | 126.3697 | 15.61781 |
| chr3 | 1.03E+08 | 1.04E+08 | 115.1524 | 14.21823 |
| chr3 | 1.2E+08 | 1.2E+08 | 105.8146 | 13.05317 |
| chr3 | 45950213 | 45999654 | 104.2006 | 12.85178 |
| chr3 | 1.2E+08 | 1.2E+08 | 103.8696 | 12.81048 |
| chr3 | 2750109 | 2799948 | 102.7639 | 12.67253 |
| chr3 | 1.18E+08 | 1.19E+08 | 95.25423 | 11.73555 |
| chr3 | 1.2E+08 | 1.2E+08 | 93.2219 | 11.48198 |
| chr3 | 1.2E+08 | 1.2E+08 | 91.87823 | 11.31433 |
| chr3 | 95450052 | 95498973 | 86.54959 | 10.64947 |
| chr3 | 1.18E+08 | 1.18E+08 | 85.77169 | 10.55242 |
| chr3 | 45900017 | 45948460 | 80.34986 | 9.875937 |
| chr3 | 95575391 | 95624884 | 79.09959 | 9.719942 |
| chr3 | 48450265 | 48499954 | 78.86875 | 9.691139 |
| chr3 | 45975417 | 46024825 | 73.74008 | 9.051238 |
| chr3 | 95650013 | 95699949 | 71.46488 | 8.767362 |
| chr4 | 8275061 | 8324969 | 108.4688 | 29.07237 |
| chr4 | 8325035 | 8374944 | 89.58855 | 23.98963 |
| chr4 | 7175163 | 7224906 | 54.47983 | 14.53805 |
| chr4 | 1925044 | 1974758 | 52.56345 | 14.02214 |
| chr4 | 2725031 | 2774967 | 49.6178 | 13.22914 |
| chr4 | 64825019 | 64874842 | 46.21494 | 12.31306 |
| chr4 | 8350031 | 8399511 | 41.80701 | 11.12641 |
| chr4 | 8300130 | 8349937 | 34.90491 | 9.268304 |
| chr4 | 75625195 | 75659779 | 27.25979 | 7.210168 |
| chr4 | 8375048 | 8424959 | 26.46352 | 6.995805 |
| chr4 | 950198 | 999699 | 24.46814 | 6.45863 |
| chr4 | 15175042 | 15224968 | 22.71345 | 5.986253 |
| chr4 | 1500105 | 1549813 | 22.01427 | 5.798027 |
| chr4 | 75500331 | 75549980 | 20.15709 | 5.298058 |
| chr4 | 14875411 | 14924925 | 19.71755 | 5.179729 |
| chr4 | 4025121 | 4074960 | 18.65955 | 4.894908 |
| chr4 | 74850012 | 74899963 | 18.16384 | 4.761459 |
| chr4 | 7125037 | 7174884 | 18.11459 | 4.748199 |
| chr4 | 7450015 | 7499973 | 16.48424 | 4.309293 |
| chr5 | 58925413 | 58974977 | 191.3625 | 24.78228 |
| chr5 | 58075133 | 58124691 | 136.2715 | 17.6054 |
| chr5 | 58900177 | 58949907 | 128.455 | 16.58713 |
| chr5 | 61300055 | 61349878 | 114.44 | 14.76136 |
| chr5 | 58050013 | 58099945 | 88.73492 | 11.41267 |
| chr5 | 58850083 | 58899990 | 88.14486 | 11.3358 |
| chr5 | 58875232 | 58924834 | 87.90275 | 11.30426 |
| chr5 | 58100104 | 58149884 | 84.00389 | 10.79635 |
| chr5 | 58550011 | 58599894 | 80.34431 | 10.3196 |
| chr5 | 57975013 | 58024952 | 64.19086 | 8.215244 |
| chr5 | 58000001 | 58049936 | 58.28402 | 7.445742 |
| chr5 | 19850300 | 19899940 | 50.23491 | 6.397161 |
| chr5 | 58575736 | 58624750 | 42.83768 | 5.433501 |
| chr5 | 62525104 | 62574956 | 42.17264 | 5.346864 |
| chr5 | 13650144 | 13699920 | 41.76679 | 5.293993 |
| chr5 | 19875423 | 19924939 | 40.29017 | 5.101629 |
| chr5 | 2900287 | 2949986 | 39.86888 | 5.046746 |
| chr5 | 57950046 | 57999953 | 36.85958 | 4.654715 |
| chr5 | 61275019 | 61324937 | 33.90295 | 4.269546 |
| chr6 | 34900205 | 34948210 | 180.7927 | 10.97631 |
| chr6 | 34925029 | 34974765 | 157.6178 | 9.517919 |
| chr6 | 12650080 | 12699602 | 134.9127 | 8.089089 |
| chr6 | 22325055 | 22374968 | 117.0565 | 6.965408 |
| chr6 | 34975737 | 35024912 | 103.3475 | 6.102704 |
| chr6 | 20575321 | 20624881 | 101.2239 | 5.969063 |
| chr6 | 21825091 | 21874753 | 99.81587 | 5.880457 |
| chr6 | 16225909 | 16274517 | 97.33756 | 5.724498 |
| chr6 | 17550240 | 17598846 | 94.54004 | 5.54845 |
| chr6 | 12700141 | 12748054 | 91.43246 | 5.352891 |
| chr6 | 16675333 | 16724771 | 87.48797 | 5.104666 |
| chr6 | 20175044 | 20224765 | 83.74213 | 4.868941 |
| chr6 | 12625595 | 12674454 | 81.58896 | 4.733442 |
| chr6 | 17600058 | 17648594 | 81.51043 | 4.7285 |
| chr6 | 17425264 | 17474580 | 79.30615 | 4.589786 |
| chr6 | 22750188 | 22799541 | 78.13593 | 4.516144 |
| chr6 | 20550605 | 20599358 | 77.37506 | 4.468263 |
| chr6 | 16250420 | 16299927 | 71.11982 | 4.074622 |
| chr6 | 20375148 | 20424790 | 70.13656 | 4.012746 |
| chr7 | 650288 | 699885 | 118.5526 | 16.1242 |
| chr7 | 38275007 | 38324523 | 80.3669 | 10.85652 |
| chr7 | 29800348 | 29849979 | 77.20543 | 10.4204 |
| chr7 | 900413 | 938581 | 68.86666 | 9.27007 |
| chr7 | 625560 | 674684 | 63.0041 | 8.461334 |
| chr7 | 10525057 | 10574829 | 57.45428 | 7.69574 |
| chr7 | 29825031 | 29874991 | 57.32349 | 7.677698 |
| chr7 | 700987 | 749679 | 49.21508 | 6.55915 |
| chr7 | 725191 | 774970 | 45.05322 | 5.985024 |
| chr7 | 800027 | 849642 | 43.22045 | 5.732195 |
| chr7 | 5775002 | 5824879 | 42.56044 | 5.641147 |
| chr7 | 10350319 | 10399716 | 41.16764 | 5.449011 |
| chr7 | 676380 | 724650 | 39.42917 | 5.209191 |
| chr7 | 425270 | 474841 | 38.26412 | 5.048473 |
| chr7 | 29776057 | 29824934 | 36.10936 | 4.751225 |
| chr7 | 38750234 | 38799876 | 36.10745 | 4.750963 |
| chr7 | 12900572 | 12949402 | 35.04267 | 4.604076 |
| chr7 | 38475443 | 38524797 | 34.62302 | 4.546186 |
| chr7 | 525830 | 574938 | 34.57351 | 4.539356 |
| chr8 | 30575050 | 30624822 | 450.6819 | 23.14149 |
| chr8 | 30500182 | 30549959 | 245.4918 | 12.53749 |
| chr8 | 30550147 | 30599942 | 240.5356 | 12.28136 |
| chr8 | 23225180 | 23274903 | 202.5393 | 10.31775 |
| chr8 | 27850035 | 27899769 | 170.7147 | 8.673094 |
| chr8 | 23250128 | 23299770 | 140.7437 | 7.124223 |
| chr8 | 30525199 | 30574710 | 102.8897 | 5.167969 |
| chr8 | 27925395 | 27974613 | 93.76227 | 4.696276 |
| chr8 | 29975136 | 30024989 | 83.25804 | 4.153429 |
| chr8 | 28000149 | 28049968 | 72.83152 | 3.614597 |
| chr8 | 30150211 | 30199941 | 69.09408 | 3.421451 |
| chr8 | 30875184 | 30924954 | 67.63894 | 3.34625 |
| chr8 | 27625838 | 27674794 | 65.87875 | 3.255286 |
| chr8 | 23325066 | 23374808 | 55.93874 | 2.741597 |
| chr8 | 25825005 | 25874768 | 53.62697 | 2.622127 |
| chr8 | 23350090 | 23399903 | 50.97903 | 2.485285 |
| chr8 | 27575092 | 27624991 | 49.91157 | 2.430119 |
| chr8 | 27950184 | 27999961 | 46.62299 | 2.260169 |
| chr8 | 16050160 | 16099875 | 44.53517 | 2.152273 |
| chr9 | 925104 | 974781 | 226.0901 | 23.7237 |
| chr9 | 17750206 | 17799983 | 94.87448 | 9.863466 |
| chr9 | 17700005 | 17749734 | 75.00267 | 7.764419 |
| chr9 | 17850535 | 17899839 | 66.75508 | 6.89323 |
| chr9 | 18425023 | 18474877 | 56.71728 | 5.832944 |
| chr9 | 1100151 | 1149614 | 55.22587 | 5.675407 |
| chr9 | 9825185 | 9874982 | 47.24629 | 4.832528 |
| chr9 | 3500093 | 3549958 | 40.37442 | 4.106656 |
| chr9 | 17450403 | 17499886 | 38.65412 | 3.924943 |
| chr9 | 12075053 | 12124976 | 36.22383 | 3.668232 |
| chr9 | 1350060 | 1399813 | 34.95732 | 3.534451 |
| chr9 | 10550057 | 10599705 | 33.02759 | 3.330615 |
| chr9 | 18450079 | 18499925 | 31.93063 | 3.214744 |
| chr9 | 17375056 | 17424749 | 31.21094 | 3.138723 |
| chr9 | 18350808 | 18399997 | 27.54729 | 2.751734 |
| chr9 | 975093 | 1024841 | 24.3344 | 2.412359 |
| chr9 | 12226055 | 12274962 | 24.33126 | 2.412027 |
| chr9 | 3900220 | 3949985 | 22.00088 | 2.165869 |
| chr9 | 17425152 | 17474927 | 21.24042 | 2.085542 |
| chr10 | 3500065 | 3549844 | 253.6576 | 15.72994 |
| chr10 | 9800209 | 9849943 | 169.019 | 10.41243 |
| chr10 | 7950102 | 7999648 | 126.404 | 7.735099 |
| chr10 | 7550466 | 7599657 | 111.3166 | 6.78722 |
| chr10 | 12200511 | 12249897 | 94.36734 | 5.722364 |
| chr10 | 6725008 | 6774877 | 92.87386 | 5.628535 |
| chr10 | 10026480 | 10074046 | 84.7481 | 5.118025 |
| chr10 | 11550420 | 11599982 | 83.51556 | 5.04059 |
| chr10 | 12225562 | 12274993 | 81.01612 | 4.88356 |
| chr10 | 10051225 | 10099612 | 79.41005 | 4.782657 |
| chr10 | 9775156 | 9824961 | 75.48187 | 4.535865 |
| chr10 | 12250849 | 12299992 | 71.32272 | 4.274562 |
| chr10 | 7825256 | 7874977 | 68.22891 | 4.080191 |
| chr10 | 12150526 | 12199872 | 60.09729 | 3.569313 |
| chr10 | 7850042 | 7899938 | 55.54334 | 3.283206 |
| chr10 | 11675017 | 11720887 | 54.78069 | 3.235293 |
| chr10 | 12000193 | 12049961 | 53.64266 | 3.163795 |
| chr10 | 12050145 | 12099830 | 49.03228 | 2.874143 |
| chr10 | 7900044 | 7949565 | 48.416 | 2.835424 |
| chr11 | 442704 | 471369 | 0.593207 | 5.670121 |
| chr11 | 701083 | 748174 | 0.096027 | 0.749539 |
| chr11 | 475001 | 520966 | 0.000717 | -0.19374 |
| chr12 | 12725046 | 12774965 | 40.39609 | 17.36744 |
| chr12 | 12050037 | 12099987 | 35.18516 | 15.10883 |
| chr12 | 1450039 | 1499997 | 24.49403 | 10.4749 |
| chr12 | 2450370 | 2499912 | 20.92176 | 8.926541 |
| chr12 | 2325122 | 2374986 | 12.88579 | 5.443451 |
| chr12 | 2350439 | 2399783 | 11.70207 | 4.930384 |
| chr12 | 3700246 | 3749960 | 8.846362 | 3.692614 |
| chr12 | 12800201 | 12849695 | 8.709716 | 3.633387 |
| chr12 | 926320 | 974607 | 7.461309 | 3.092281 |
| chr12 | 3775377 | 3824556 | 4.626806 | 1.863702 |
| chr12 | 7980386 | 8024565 | 4.26802 | 1.708191 |
| chr12 | 750063 | 799804 | 3.842208 | 1.523629 |
| chr12 | 12775042 | 12824901 | 3.747668 | 1.482651 |
| chr12 | 2275095 | 2324816 | 3.255851 | 1.269479 |
| chr12 | 2400515 | 2449823 | 3.228037 | 1.257424 |
| chr12 | 5300200 | 5349999 | 2.908059 | 1.118733 |
| chr12 | 20875107 | 20924984 | 2.897018 | 1.113948 |
| chr12 | 12325019 | 12374911 | 2.86602 | 1.100512 |
| chr12 | 7050020 | 7099730 | 2.791415 | 1.068176 |
| chr13 | 5400009 | 5449946 | 255.3599 | 17.21651 |
| chr13 | 5025179 | 5074856 | 144.5358 | 9.669522 |
| chr13 | 5050008 | 5099694 | 132.5728 | 8.854854 |
| chr13 | 5300200 | 5349961 | 110.0092 | 7.3183 |
| chr13 | 5100033 | 5149827 | 98.02745 | 6.502353 |
| chr13 | 5000123 | 5049995 | 86.03765 | 5.685861 |
| chr13 | 5375088 | 5424967 | 85.8223 | 5.671196 |
| chr13 | 5350148 | 5399970 | 83.35521 | 5.50319 |
| chr13 | 19400024 | 19449997 | 77.1385 | 5.079839 |
| chr13 | 650421 | 699905 | 76.7316 | 5.05213 |
| chr13 | 775042 | 824488 | 72.8616 | 4.788587 |
| chr13 | 7350113 | 7399802 | 66.35526 | 4.345512 |
| chr13 | 625367 | 674545 | 66.09386 | 4.327712 |
| chr13 | 5150237 | 5199935 | 62.82548 | 4.105138 |
| chr13 | 5075259 | 5124945 | 55.174 | 3.584081 |
| chr13 | 4875113 | 4924791 | 52.01186 | 3.368743 |
| chr13 | 5200148 | 5249533 | 45.10674 | 2.898512 |
| chr13 | 4975011 | 5024798 | 42.10623 | 2.694181 |
| chr13 | 5325022 | 5374286 | 39.93249 | 2.546152 |
| chr14 | 16000055 | 16049990 | 240.2934 | 10.22663 |
| chr14 | 7000281 | 7049879 | 208.3162 | 8.83925 |
| chr14 | 6900413 | 6949920 | 199.3396 | 8.449783 |
| chr14 | 6875123 | 6924827 | 182.6479 | 7.725586 |
| chr14 | 11600331 | 11649904 | 179.0993 | 7.571624 |
| chr14 | 16025107 | 16074952 | 174.4406 | 7.3695 |
| chr14 | 6950357 | 6999864 | 171.0312 | 7.221576 |
| chr14 | 16075050 | 16124989 | 167.2383 | 7.057017 |
| chr14 | 16050046 | 16099882 | 150.7792 | 6.342911 |
| chr14 | 6925046 | 6974524 | 149.9483 | 6.306863 |
| chr14 | 11525048 | 11574727 | 122.1954 | 5.102754 |
| chr14 | 6975532 | 7024891 | 104.361 | 4.328979 |
| chr14 | 11550062 | 11599809 | 95.91762 | 3.962651 |
| chr14 | 16125168 | 16174973 | 93.7238 | 3.867469 |
| chr14 | 21550618 | 21599830 | 86.10719 | 3.53701 |
| chr14 | 11575172 | 11624964 | 83.07037 | 3.405252 |
| chr14 | 21525130 | 21574439 | 81.70335 | 3.345942 |
| chr14 | 6825148 | 6874678 | 79.34439 | 3.243594 |
| chr14 | 15975385 | 16024785 | 75.38463 | 3.071794 |
| chr15 | 4825136 | 4874932 | 361.5895 | 19.01193 |
| chr15 | 4800106 | 4849722 | 202.2885 | 10.58744 |
| chr15 | 4750194 | 4799991 | 190.9515 | 9.987893 |
| chr15 | 1125003 | 1174979 | 112.7176 | 5.850565 |
| chr15 | 12125170 | 12174881 | 100.8142 | 5.221065 |
| chr15 | 1075104 | 1124502 | 100.4954 | 5.204206 |
| chr15 | 1100065 | 1149401 | 78.55908 | 4.044125 |
| chr15 | 4725176 | 4774956 | 71.63018 | 3.677697 |
| chr15 | 4875684 | 4924976 | 68.8327 | 3.529755 |
| chr15 | 4701770 | 4749388 | 36.52532 | 1.821209 |
| chr15 | 4775121 | 4824384 | 16.59193 | 0.76705 |
| chr15 | 3901071 | 3949966 | 12.59682 | 0.555772 |
| chr15 | 11525241 | 11573206 | 10.03788 | 0.420445 |
| chr15 | 17125340 | 17174604 | 8.220987 | 0.32436 |
| chr15 | 12925350 | 12974702 | 7.316663 | 0.276536 |
| chr15 | 13900016 | 13949894 | 6.925756 | 0.255863 |
| chr15 | 1775043 | 1824937 | 6.178697 | 0.216356 |
| chr15 | 3800064 | 3849868 | 5.599709 | 0.185736 |
| chr15 | 4850137 | 4899881 | 5.168269 | 0.16292 |
| chr16 | 875026 | 924994 | 73.49934 | 18.32977 |
| chr16 | 15100155 | 15149697 | 38.32647 | 9.476617 |
| chr16 | 850324 | 899801 | 33.82787 | 8.344301 |
| chr16 | 4475220 | 4524981 | 26.01783 | 6.378482 |
| chr16 | 8425183 | 8474797 | 24.37342 | 5.964577 |
| chr16 | 14150954 | 14199146 | 21.2428 | 5.176587 |
| chr16 | 14175140 | 14224985 | 11.20847 | 2.650908 |
| chr16 | 13375135 | 13424981 | 10.73376 | 2.531422 |
| chr16 | 15125098 | 15174382 | 8.931308 | 2.077737 |
| chr16 | 9750262 | 9799737 | 8.760135 | 2.034652 |
| chr16 | 3900066 | 3949975 | 6.94168 | 1.576939 |
| chr16 | 13400141 | 13449977 | 6.783729 | 1.537182 |
| chr16 | 2700167 | 2749837 | 6.431403 | 1.4485 |
| chr16 | 8875598 | 8924694 | 6.299618 | 1.41533 |
| chr16 | 11025321 | 11074587 | 6.223133 | 1.396078 |
| chr16 | 9275104 | 9324954 | 6.065402 | 1.356376 |
| chr16 | 8450171 | 8499804 | 5.625642 | 1.245687 |
| chr16 | 3675119 | 3724853 | 5.146734 | 1.125144 |
| chr16 | 14725208 | 14774366 | 4.559082 | 0.97723 |
| chr17 | 1450944 | 1475722 | 3.416339 | 7.912421 |
| chr17 | 1401755 | 1449275 | 0.498746 | 1.02891 |
| chr17 | 614586 | 648440 | 0.094485 | 0.075133 |
| chr17 | 1250170 | 1299262 | 0.057339 | -0.01251 |
| chr17 | 692939 | 703839 | 0.004421 | -0.13736 |
| chr17 | 1425003 | 1457535 | 0.000227 | -0.14725 |
| chr17 | 890736 | 924809 | 3.80E-05 | -0.1477 |
| chr18 | 5800169 | 5849955 | 59.04069 | 9.487057 |
| chr18 | 7675162 | 7724939 | 55.94304 | 8.977872 |
| chr18 | 7825303 | 7874956 | 50.01405 | 8.003281 |
| chr18 | 6400014 | 6449630 | 45.09516 | 7.194726 |
| chr18 | 7150375 | 7199875 | 34.15604 | 5.396581 |
| chr18 | 5825075 | 5874970 | 33.49597 | 5.288081 |
| chr18 | 6501352 | 6549911 | 32.55746 | 5.133811 |
| chr18 | 8075029 | 8124912 | 26.17006 | 4.083867 |
| chr18 | 7775443 | 7824961 | 24.11839 | 3.74662 |
| chr18 | 4225034 | 4274995 | 23.0458 | 3.57031 |
| chr18 | 2825163 | 2874833 | 20.63279 | 3.173665 |
| chr18 | 6450113 | 6499509 | 17.33854 | 2.632165 |
| chr18 | 5750213 | 5799886 | 16.05978 | 2.421966 |
| chr18 | 2775194 | 2824741 | 12.84627 | 1.893737 |
| chr18 | 10825036 | 10874860 | 10.69771 | 1.540562 |
| chr18 | 2725307 | 2774848 | 10.62456 | 1.528539 |
| chr18 | 7800064 | 7849641 | 10.53645 | 1.514055 |
| chr18 | 7900159 | 7949961 | 9.034491 | 1.267167 |
| chr18 | 11500430 | 11549865 | 7.992375 | 1.095867 |
| chr19 | 5950027 | 5999911 | 58.32822 | 12.71445 |
| chr19 | 5825022 | 5873813 | 51.99246 | 11.3105 |
| chr19 | 5050032 | 5099929 | 37.74512 | 8.153387 |
| chr19 | 5025006 | 5074940 | 34.03555 | 7.331373 |
| chr19 | 400902 | 449978 | 17.88784 | 3.753157 |
| chr19 | 6450020 | 6499664 | 15.97332 | 3.328914 |
| chr19 | 10650021 | 10699911 | 15.89988 | 3.312641 |
| chr19 | 11750131 | 11799916 | 14.11738 | 2.917652 |
| chr19 | 7950100 | 7999981 | 13.47847 | 2.776074 |
| chr19 | 11500151 | 11549512 | 12.99204 | 2.668285 |
| chr19 | 5600014 | 5649580 | 11.92966 | 2.432868 |
| chr19 | 7500169 | 7549661 | 11.13858 | 2.257571 |
| chr19 | 7525021 | 7574818 | 10.51049 | 2.11839 |
| chr19 | 10675007 | 10724996 | 9.737114 | 1.947016 |
| chr19 | 11675049 | 11724875 | 8.216159 | 1.609983 |
| chr19 | 4825119 | 4874793 | 8.200893 | 1.6066 |
| chr19 | 6400022 | 6449983 | 8.066307 | 1.576777 |
| chr19 | 5901131 | 5949814 | 7.119458 | 1.366962 |
| chr19 | 4875073 | 4924934 | 6.820396 | 1.300692 |
| chr20 | 6125141 | 6174870 | 84.73321 | 10.98147 |
| chr20 | 5351599 | 5399509 | 80.81127 | 10.46613 |
| chr20 | 6075087 | 6124876 | 58.91082 | 7.5884 |
| chr20 | 3000088 | 3049666 | 25.88048 | 3.248201 |
| chr20 | 2975055 | 3024991 | 22.5815 | 2.814714 |
| chr20 | 7750012 | 7799928 | 10.50616 | 1.228009 |
| chr20 | 2950072 | 2999293 | 8.319376 | 0.940666 |
| chr20 | 6100251 | 6149946 | 7.190458 | 0.792325 |
| chr20 | 7400123 | 7449954 | 6.416289 | 0.690599 |
| chr20 | 4600035 | 4649865 | 4.325128 | 0.41582 |
| chr20 | 7675078 | 7724993 | 3.632529 | 0.324812 |
| chr20 | 7350372 | 7399984 | 3.628119 | 0.324233 |
| chr20 | 2850013 | 2899800 | 3.605051 | 0.321202 |
| chr20 | 7525107 | 7574913 | 3.396764 | 0.293833 |
| chr20 | 6050010 | 6099880 | 3.118043 | 0.257208 |
| chr20 | 6025143 | 6074976 | 2.908873 | 0.229724 |
| chr20 | 6925097 | 6974991 | 2.873494 | 0.225075 |
| chr20 | 4850268 | 4899589 | 2.843529 | 0.221137 |
| chr20 | 2925059 | 2974365 | 2.804121 | 0.215959 |
| chr21 | 3050140 | 3099861 | 76.95947 | 23.29084 |
| chr21 | 12825088 | 12874937 | 18.39238 | 5.468589 |
| chr21 | 11875080 | 11924756 | 13.34452 | 3.932499 |
| chr21 | 10975044 | 11024365 | 12.12787 | 3.562268 |
| chr21 | 3825039 | 3874942 | 8.716327 | 2.524118 |
| chr21 | 8550465 | 8599973 | 7.56357 | 2.173328 |
| chr21 | 10125045 | 10174493 | 6.185978 | 1.75412 |
| chr21 | 2625030 | 2674978 | 5.752133 | 1.622099 |
| chr21 | 14500009 | 14549929 | 5.268426 | 1.474904 |
| chr21 | 12650237 | 12699946 | 4.76536 | 1.321819 |
| chr21 | 10250720 | 10299858 | 4.734116 | 1.312311 |
| chr21 | 10550084 | 10599583 | 4.285769 | 1.175877 |
| chr21 | 5751024 | 5799948 | 4.117139 | 1.124562 |
| chr21 | 8200058 | 8249886 | 4.06738 | 1.10942 |
| chr21 | 14825019 | 14874923 | 3.840014 | 1.040231 |
| chr21 | 8225158 | 8274934 | 3.833467 | 1.038239 |
| chr21 | 9450269 | 9499915 | 2.926617 | 0.76228 |
| chr21 | 4825261 | 4874647 | 2.891206 | 0.751505 |
| chr21 | 2175057 | 2224668 | 2.788459 | 0.720238 |
| chr22 | 2025130 | 2074828 | 128.6892 | 10.77581 |
| chr22 | 2050004 | 2099489 | 117.444 | 9.818334 |
| chr22 | 7775066 | 7824872 | 87.8207 | 7.296067 |
| chr22 | 2000050 | 2049994 | 66.97864 | 5.521475 |
| chr22 | 7225391 | 7274980 | 53.82641 | 4.401632 |
| chr22 | 2450497 | 2499898 | 32.15941 | 2.5568 |
| chr22 | 7175132 | 7224871 | 29.49974 | 2.330343 |
| chr22 | 7800002 | 7849933 | 28.17272 | 2.217355 |
| chr22 | 6775020 | 6824811 | 20.53724 | 1.567233 |
| chr22 | 7025089 | 7074727 | 17.43527 | 1.303117 |
| chr22 | 7150149 | 7199986 | 16.48959 | 1.222597 |
| chr22 | 6225053 | 6274976 | 12.04022 | 0.843757 |
| chr22 | 6875035 | 6924977 | 11.557 | 0.802613 |
| chr22 | 6850082 | 6899926 | 11.15222 | 0.768148 |
| chr22 | 1975198 | 2024916 | 9.920551 | 0.663278 |
| chr22 | 6650073 | 6699985 | 7.350682 | 0.444467 |
| chr22 | 825570 | 874669 | 6.916674 | 0.407514 |
| chr22 | 7375234 | 7424995 | 6.302122 | 0.355188 |
| chr22 | 6450162 | 6499968 | 5.86761 | 0.318191 |
| chr23 | 6150372 | 6199880 | 9.437642 | 10.51414 |
| chr23 | 5750893 | 5799823 | 7.841339 | 8.704026 |
| chr23 | 1525002 | 1574819 | 4.929059 | 5.401672 |
| chr23 | 4650033 | 4699998 | 3.091981 | 3.318532 |
| chr23 | 1025027 | 1074862 | 3.072661 | 3.296624 |
| chr23 | 4675014 | 4724916 | 1.882947 | 1.947558 |
| chr23 | 4500363 | 4543121 | 1.606666 | 1.634271 |
| chr23 | 4050213 | 4099915 | 1.273146 | 1.256079 |
| chr23 | 2275047 | 2323643 | 0.913491 | 0.848251 |
| chr23 | 1400358 | 1448960 | 0.888214 | 0.819589 |
| chr23 | 4700244 | 4749970 | 0.776574 | 0.692996 |
| chr23 | 1100254 | 1149518 | 0.697797 | 0.603667 |
| chr23 | 2450125 | 2499800 | 0.650117 | 0.549601 |
| chr23 | 902818 | 949670 | 0.616067 | 0.51099 |
| chr23 | 5200078 | 5249934 | 0.552349 | 0.438738 |
| chr23 | 2725076 | 2774972 | 0.545968 | 0.431502 |
| chr23 | 3000046 | 3049894 | 0.545809 | 0.431322 |
| chr23 | 1750030 | 1799982 | 0.450889 | 0.323687 |
| chr23 | 4250093 | 4299494 | 0.422851 | 0.291894 |
| chr24 | 3025030 | 3074907 | 57.02208 | 11.30639 |
| chr24 | 4850033 | 4899906 | 51.22075 | 10.13687 |
| chr24 | 5425049 | 5474947 | 24.34608 | 4.719042 |
| chr24 | 6975106 | 7023972 | 23.48708 | 4.545873 |
| chr24 | 7000639 | 7049984 | 12.76839 | 2.385026 |
| chr24 | 7250763 | 7297830 | 11.68221 | 2.166058 |
| chr24 | 2875024 | 2924962 | 8.810794 | 1.587191 |
| chr24 | 5725044 | 5774934 | 8.324771 | 1.489211 |
| chr24 | 3425186 | 3472495 | 7.890675 | 1.401699 |
| chr24 | 7175160 | 7224383 | 6.288009 | 1.078608 |
| chr24 | 5626324 | 5674602 | 6.046218 | 1.029864 |
| chr24 | 7400037 | 7447932 | 5.793804 | 0.978978 |
| chr24 | 600039 | 649870 | 4.548103 | 0.72785 |
| chr24 | 5675048 | 5724975 | 4.159104 | 0.649429 |
| chr24 | 1525589 | 1574919 | 3.454856 | 0.507455 |
| chr24 | 6000092 | 6049819 | 3.285527 | 0.473319 |
| chr24 | 4200129 | 4249993 | 2.866354 | 0.388816 |
| chr24 | 7050350 | 7099823 | 2.813285 | 0.378117 |
| chr24 | 4800024 | 4849942 | 2.537849 | 0.322591 |
| chr25 | 1675092 | 1724933 | 91.57077 | 11.19684 |
| chr25 | 2875098 | 2924969 | 75.89925 | 9.251505 |
| chr25 | 1600253 | 1649769 | 45.45684 | 5.472631 |
| chr25 | 3100128 | 3149938 | 40.70272 | 4.882494 |
| chr25 | 7400060 | 7448807 | 40.62211 | 4.872487 |
| chr25 | 3775379 | 3824844 | 33.00894 | 3.927449 |
| chr25 | 1575032 | 1624753 | 12.05617 | 1.326543 |
| chr25 | 7200046 | 7249495 | 11.81817 | 1.297 |
| chr25 | 1000191 | 1049823 | 8.467298 | 0.88105 |
| chr25 | 1300012 | 1349755 | 7.989454 | 0.821734 |
| chr25 | 2950066 | 2999635 | 5.676699 | 0.534647 |
| chr25 | 1550705 | 1599455 | 5.44012 | 0.50528 |
| chr25 | 7125222 | 7174753 | 5.298117 | 0.487653 |
| chr25 | 3800135 | 3849859 | 4.280991 | 0.361395 |
| chr25 | 850062 | 899827 | 3.745931 | 0.294977 |
| chr25 | 7350044 | 7399702 | 3.717846 | 0.291491 |
| chr25 | 7325425 | 7374928 | 3.408149 | 0.253048 |
| chr25 | 7000346 | 7049991 | 3.27503 | 0.236523 |
| chr25 | 4750105 | 4799967 | 2.63687 | 0.157307 |
| chr26 | 728750 | 771267 | 67.3598 | 7.830152 |
| chr26 | 1676488 | 1724969 | 54.12129 | 6.247898 |
| chr26 | 751191 | 799978 | 33.78027 | 3.816759 |
| chr26 | 700017 | 748514 | 20.72173 | 2.256016 |
| chr26 | 987935 | 1024762 | 10.11341 | 0.988119 |
| chr26 | 1125016 | 1168938 | 7.163681 | 0.635571 |
| chr26 | 2000001 | 2046248 | 4.961276 | 0.372342 |
| chr26 | 2400402 | 2449962 | 4.486161 | 0.315557 |
| chr26 | 325003 | 374860 | 4.39996 | 0.305254 |
| chr26 | 875350 | 924771 | 4.389923 | 0.304054 |
| chr26 | 478026 | 524924 | 3.954738 | 0.252041 |
| chr26 | 1176963 | 1221694 | 3.29622 | 0.173336 |
| chr26 | 600037 | 648948 | 2.541016 | 0.083075 |
| chr26 | 675707 | 724064 | 1.998287 | 0.018208 |
| chr26 | 2275545 | 2324876 | 1.818985 | -0.00322 |
| chr26 | 1975775 | 2024877 | 1.506921 | -0.04052 |
| chr26 | 2200147 | 2249974 | 1.451485 | -0.04714 |
| chr26 | 650265 | 699858 | 1.34355 | -0.06005 |
| chr26 | 2300018 | 2335716 | 1.083092 | -0.09117 |
| chr27 | 7400544 | 7449917 | 16.59726 | 9.735226 |
| chr27 | 4425048 | 4474861 | 15.74459 | 9.221495 |
| chr27 | 6175223 | 6224983 | 10.83714 | 6.264787 |
| chr27 | 1650328 | 1699835 | 6.384645 | 3.582189 |
| chr27 | 1900129 | 1949858 | 5.932206 | 3.309598 |
| chr27 | 6275066 | 6324961 | 5.743826 | 3.1961 |
| chr27 | 6200077 | 6249764 | 5.730478 | 3.188058 |
| chr27 | 5700490 | 5749993 | 5.301106 | 2.929364 |
| chr27 | 2680710 | 2724889 | 5.156572 | 2.842283 |
| chr27 | 6625030 | 6674913 | 3.462483 | 1.821606 |
| chr27 | 5525247 | 5574995 | 3.389634 | 1.777715 |
| chr27 | 3075010 | 3124870 | 3.240486 | 1.687854 |
| chr27 | 3550214 | 3599936 | 3.233422 | 1.683598 |
| chr27 | 4475010 | 4524963 | 3.18406 | 1.653858 |
| chr27 | 5400043 | 5449513 | 2.8688 | 1.463916 |
| chr27 | 4500039 | 4549831 | 2.853506 | 1.454701 |
| chr27 | 2451039 | 2499876 | 2.672535 | 1.345667 |
| chr27 | 7225088 | 7274892 | 2.646627 | 1.330058 |
| chr27 | 2875183 | 2924696 | 2.535189 | 1.262917 |
| chr28 | 1550216 | 1599939 | 65.68863 | 11.9411 |
| chr28 | 700054 | 749204 | 34.47898 | 6.178363 |
| chr28 | 1025121 | 1074533 | 26.46734 | 4.699046 |
| chr28 | 578467 | 624793 | 25.73926 | 4.564608 |
| chr28 | 650008 | 699719 | 18.11112 | 3.156103 |
| chr28 | 4275237 | 4306266 | 10.5338 | 1.756981 |
| chr28 | 726736 | 774287 | 6.51921 | 1.015702 |
| chr28 | 1425082 | 1474763 | 6.052413 | 0.92951 |
| chr28 | 1525030 | 1574899 | 4.981713 | 0.731809 |
| chr28 | 600042 | 649593 | 4.840445 | 0.705725 |
| chr28 | 3325047 | 3374953 | 4.620047 | 0.665029 |
| chr28 | 2325436 | 2374860 | 3.387722 | 0.437485 |
| chr28 | 2275024 | 2324994 | 3.325861 | 0.426063 |
| chr28 | 1450319 | 1499861 | 2.957572 | 0.35806 |
| chr28 | 5400058 | 5449755 | 2.380544 | 0.251514 |
| chr28 | 3600124 | 3649939 | 2.300598 | 0.236752 |
| chr28 | 1978936 | 2024992 | 2.036013 | 0.187898 |
| chr28 | 4050008 | 4099778 | 1.770179 | 0.138812 |
| chr28 | 1000358 | 1049764 | 1.74315 | 0.133822 |
| chr29 | 4501166 | 4549627 | 82.4666 | 15.01111 |
| chr29 | 4652727 | 4699955 | 16.91092 | 2.975536 |
| chr29 | 4575074 | 4623440 | 9.727385 | 1.656688 |
| chr29 | 4600041 | 4647713 | 8.279333 | 1.390836 |
| chr29 | 1500093 | 1549900 | 6.947641 | 1.146346 |
| chr29 | 825236 | 873561 | 5.934501 | 0.960341 |
| chr29 | 1085598 | 1124522 | 5.146856 | 0.815735 |
| chr29 | 1850103 | 1899772 | 3.047234 | 0.430259 |
| chr29 | 4100002 | 4149654 | 2.813987 | 0.387436 |
| chr29 | 1000068 | 1049987 | 2.637346 | 0.355006 |
| chr29 | 1975107 | 2024593 | 2.410302 | 0.313322 |
| chr29 | 4700302 | 4749990 | 2.278337 | 0.289094 |
| chr29 | 3400100 | 3449975 | 1.793439 | 0.20007 |
| chr29 | 4900733 | 4949751 | 1.603274 | 0.165157 |
| chr29 | 5877109 | 5924995 | 1.596937 | 0.163994 |
| chr29 | 4150052 | 4199982 | 1.451453 | 0.137284 |
| chr29 | 2925018 | 2974997 | 1.420186 | 0.131544 |
| chr29 | 925560 | 974935 | 1.334717 | 0.115852 |
| chr29 | 2950002 | 2999976 | 1.194729 | 0.090151 |

**Supplementary Figures**
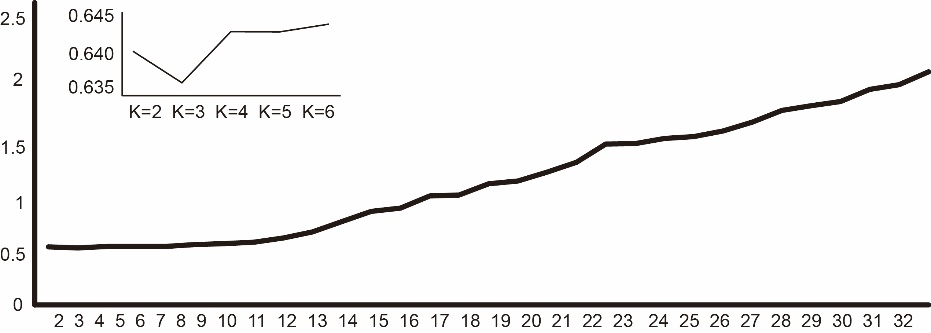


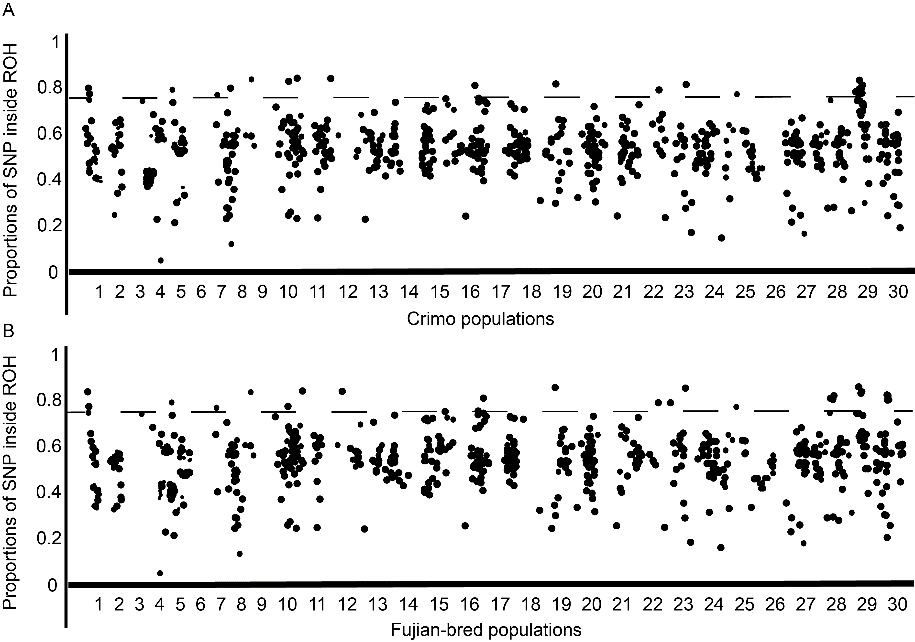
Figure S1. The correlation curve between k-value and the cross-validation error

Figure S2. The proportion of ROH hotspots across genome. Each spot shows one hotspot .(A) ROH islands in Crimo populations (B) ROH islands in Fujian-bred populations


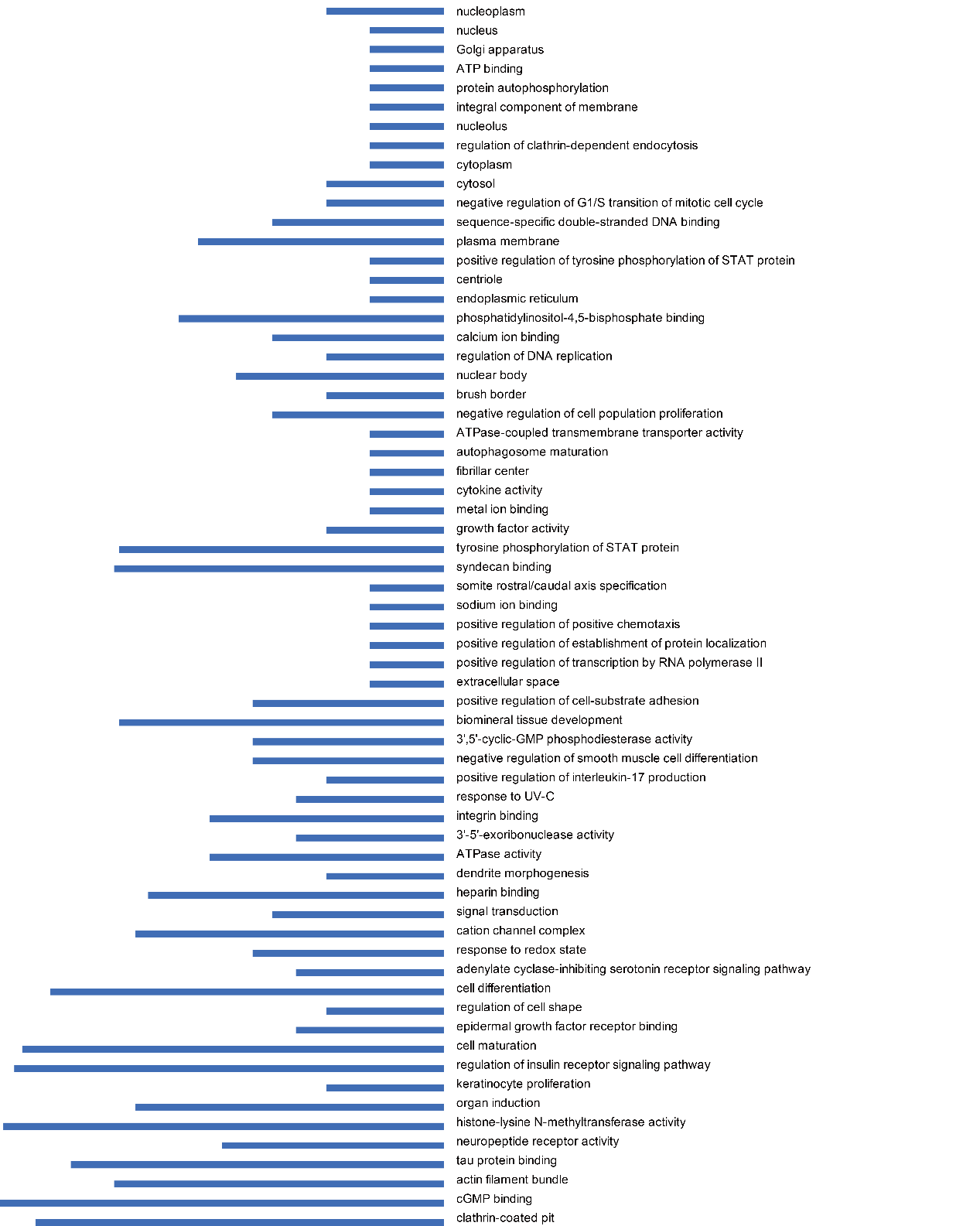


Figure S3 The enrichment analysis of GO enrichment analyses of ROH Fragments in Commercial populations


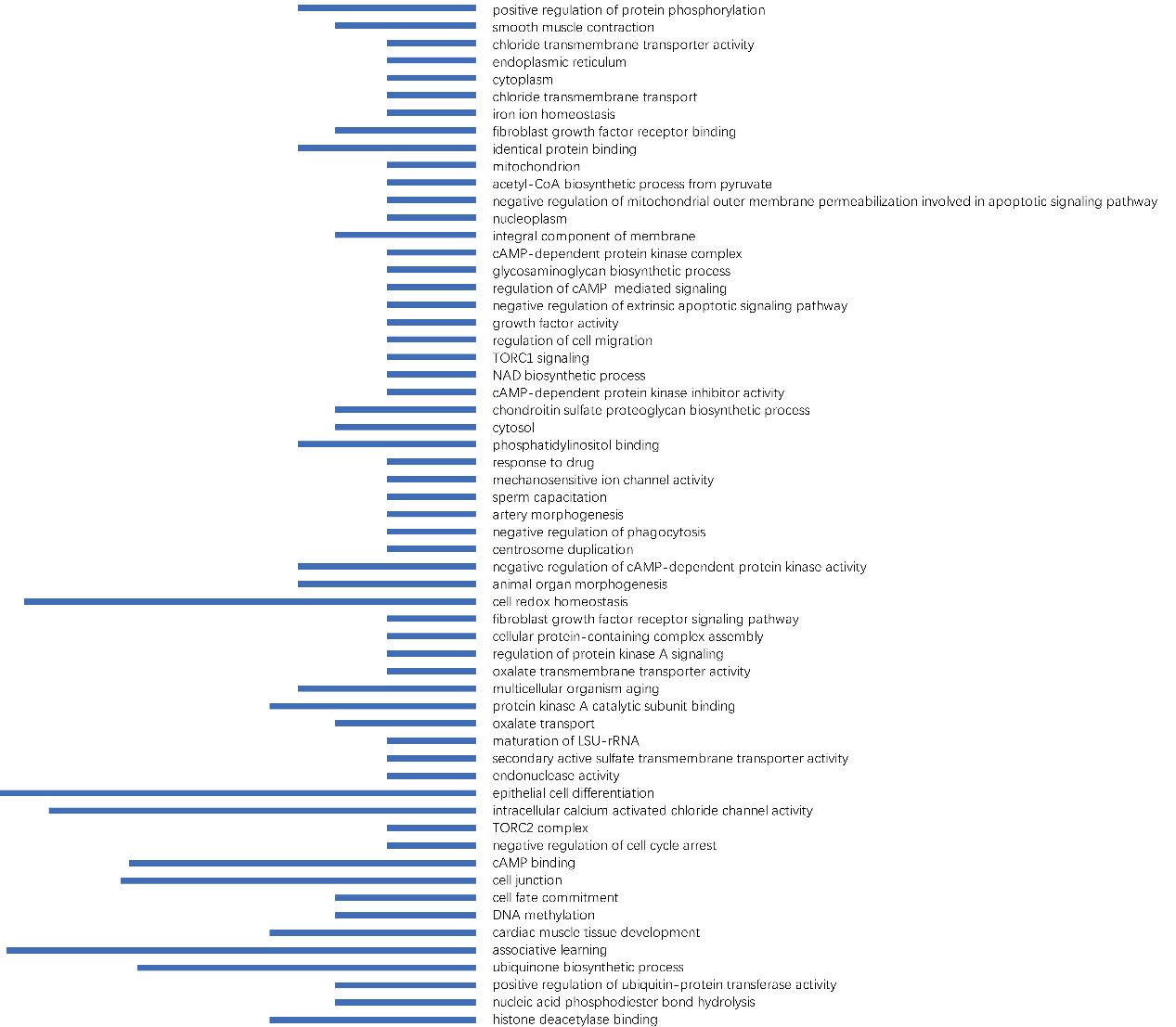


Figure S4 The enrichment analysis of GO enrichment analyses of ROH Fragments in Fujian-bred populations


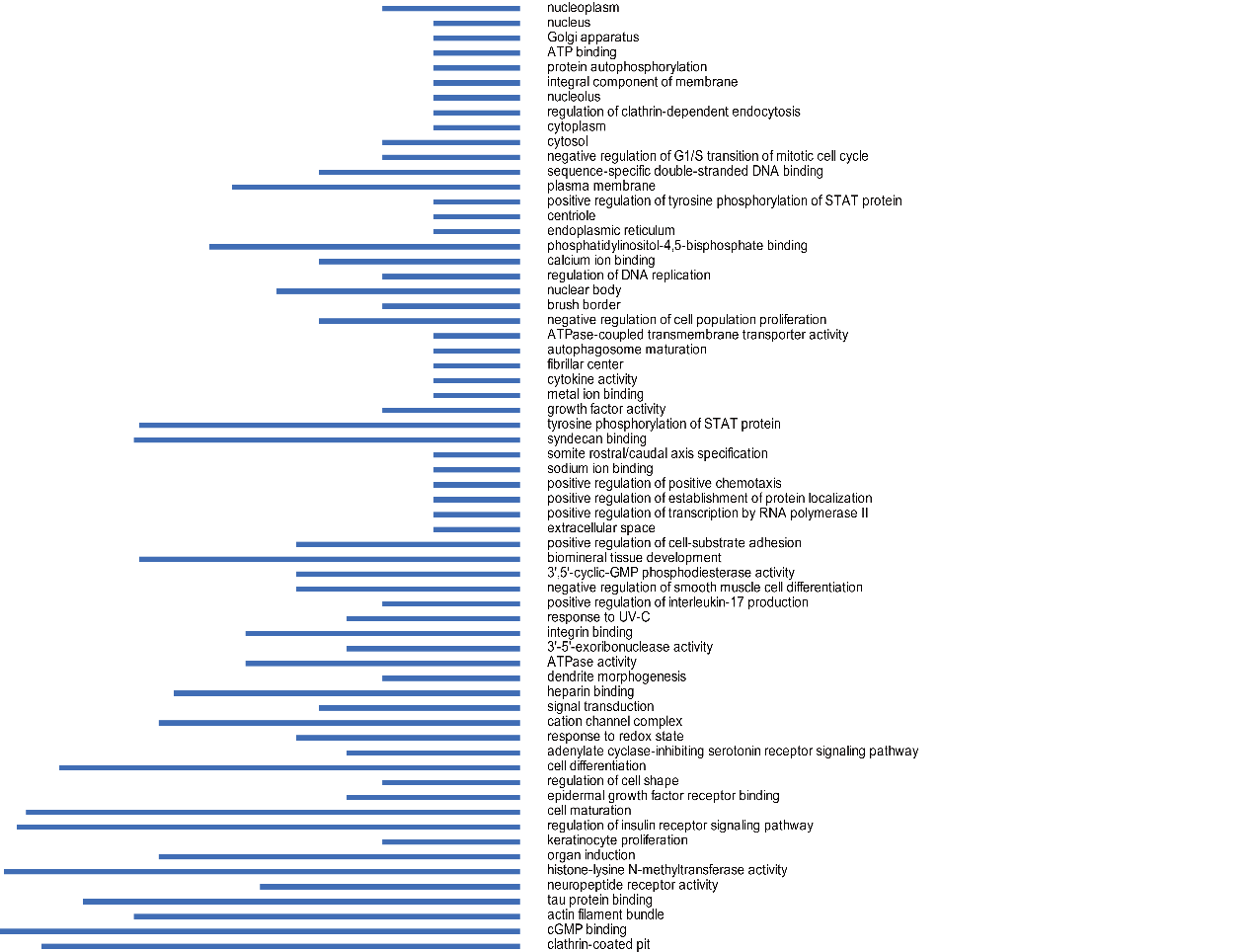


Figure S5 The enrichment analysis of GO enrichment analyses of HBD and ROH Fragments in Commercial populations


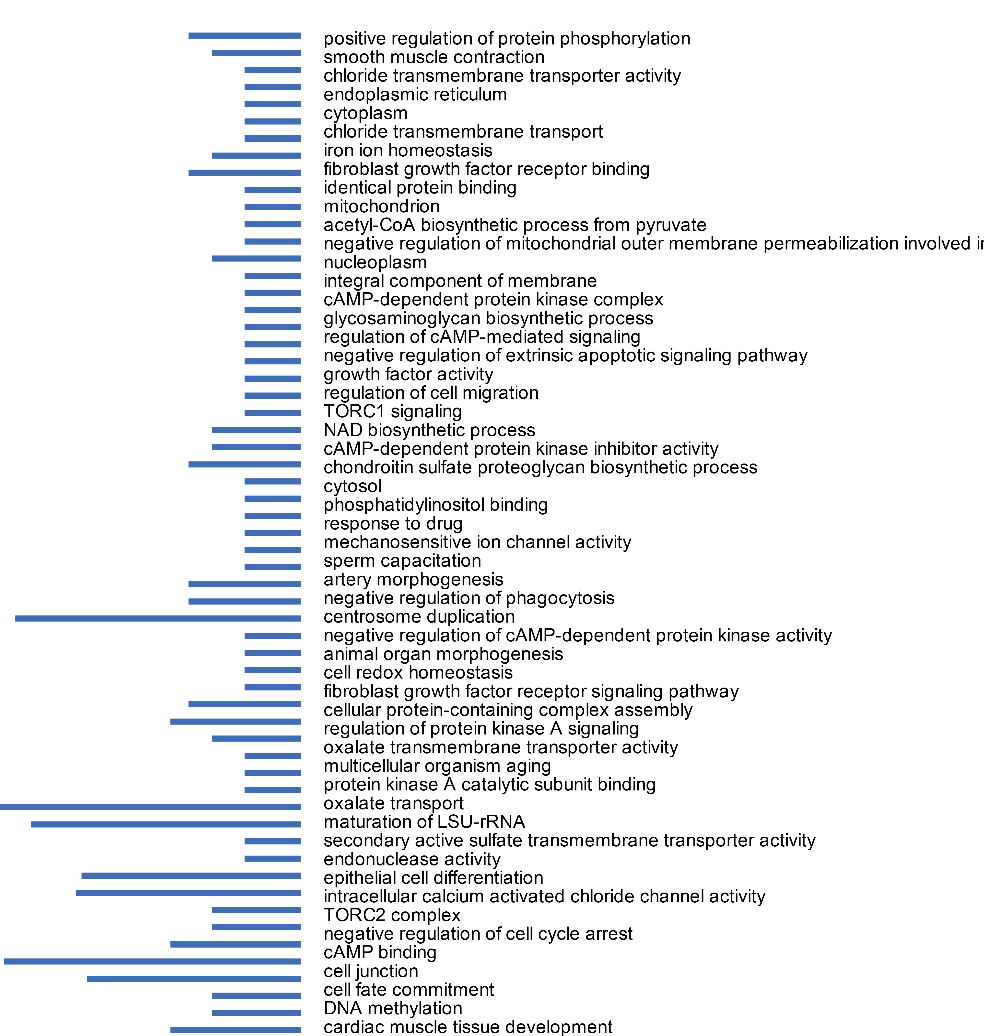


Figure S6 The enrichment analysis of GO enrichment analyses of HBD and ROH Fragments in Fujian-bred populations
